# Glioma cells require one-carbon metabolism to survive glutamine starvation

**DOI:** 10.1186/s40478-020-01114-1

**Published:** 2021-01-19

**Authors:** Kazuhiro Tanaka, Takashi Sasayama, Hiroaki Nagashima, Yasuhiro Irino, Masatomo Takahashi, Yoshihiro Izumi, Takiko Uno, Naoko Satoh, Akane Kitta, Katsusuke Kyotani, Yuichi Fujita, Mitsuru Hashiguchi, Tomoaki Nakai, Masaaki Kohta, Yoichi Uozumi, Masakazu Shinohara, Kohkichi Hosoda, Takeshi Bamba, Eiji Kohmura

**Affiliations:** 1 Department of Neurosurgery, Kobe University Graduate School of Medicine and Kobe University Hospital, 7-5-1, Kusunoki-cho, Chuo-ku, Kobe, 650-0017 Japan; 2 Division of Evidence-Based Laboratory Medicine, Kobe University Graduate School of Medicine, Kobe, 650-0017 Japan; 3 The Integrated Center for Mass Spectrometry, Kobe University Graduate School of Medicine, Kobe, 650-0017 Japan; 4 Division of Epidemiology, Kobe University Graduate School of Medicine and Kobe University Hospital, Kobe, 650-0017 Japan; 5 Department of Pathology, Kobe University Graduate School of Medicine and Kobe University Hospital, Kobe, 650-0017 Japan; 6 Center for Radiology and Radiation Oncology, Kobe University Graduate School of Medicine and Kobe University Hospital, Kobe, 650-0017 Japan; 7 Department of Neurosurgery, Kobe City Nishi-Kobe Medical Center, Kobe, 651-2273 Japan; 8 Division of Metabolomics, Medical Institute of Bioregulation, Kyushu University, Fukuoka, 812-8582 Japan

**Keywords:** One-carbon metabolism, Serine synthesis, Glutamine starvation, Glioblastoma multiforme

## Abstract

Cancer cells optimize nutrient utilization to supply energetic and biosynthetic pathways. This metabolic process also includes redox maintenance and epigenetic regulation through nucleic acid and protein methylation, which enhance tumorigenicity and clinical resistance. However, less is known about how cancer cells exhibit metabolic flexibility to sustain cell growth and survival from nutrient starvation. Here, we find that serine and glycine levels were higher in low-nutrient regions of tumors in glioblastoma multiforme (GBM) patients than they were in other regions. Metabolic and functional studies in GBM cells demonstrated that serine availability and one-carbon metabolism support glioma cell survival following glutamine deprivation. Serine synthesis was mediated through autophagy rather than glycolysis. Gene expression analysis identified upregulation of methylenetetrahydrofolate dehydrogenase 2 (MTHFD2) to regulate one-carbon metabolism. In clinical samples, MTHFD2 expression was highest in the nutrient-poor areas around “pseudopalisading necrosis.” Genetic suppression of MTHFD2 and autophagy inhibition caused tumor cell death and growth inhibition of glioma cells upon glutamine deprivation. These results highlight a critical role for serine-dependent one-carbon metabolism in surviving glutamine starvation and suggest new therapeutic targets for glioma cells adapting to a low-nutrient microenvironment.

**Electronic supplementary material:**

The online version of this article (10.1186/s40478-020-01114-1) contains supplementary material, which is available to authorized users.

## Introduction

Cancer cells must take up glucose, amino acids and lipids at an accelerated rate to support growth and energy production [8]. These nutrients are delivered by the vasculature and are transported by various transporters that are commonly upregulated across many cancers. However, tumor blood vessels are weak, leaky and fragile [9]. Abnormal vasculature combined with the high interstitial pressure within the tumor can severely compromise nutrient delivery to tumor cells, promoting metabolic heterogeneity within the tumor. Amino acids and glucose are depleted in the core of tumors relative to normal tissues, particularly in poorly vascularized areas. In addition, increased nutrient demand and uptake by tumor cells may further reduce extracellular nutrient levels. Most nutrient-deprived cells might be destined for necrotic death, while some subpopulations, such as transiently hypoxic and low-nutrient cells, might also develop malignancy and therapeutic resistance [12]. Tumor metabolic plasticity may facilitate the adaptation of cancer cells to a low-nutrient microenvironment. Uncovering molecular and metabolic responses to nutrient starvation can provide precious insights into identifying new drug targets for malignant glioma therapies.

During nutrient starvation, cancer cells can use autophagy-mediated recycling to maintain mitochondrial function and energy homeostasis to meet the elevated metabolic demand of growth and proliferation [40]. Glutamine starvation activates the general amino acid control pathway to increase amino acid uptake [5]. In particular, serine is required for a number of biosynthetic and signaling pathways, including the synthesis of other amino acids, such as glycine and cysteine, and the production of phospholipids, such as sphingolipids and phosphatidylserine [41]. Serine is also a major donor of one carbon units to the folate cycle through one-carbon metabolism. One-carbon metabolism, involving the folate and methionine cycles, integrates nutritional status from amino acids, glucose and vitamins and generates diverse outputs, such as the biosynthesis of lipids, nucleotides and proteins; the maintenance of redox status; and the substrates for methylation reactions [16]. In leukemia cells, glutamine deprivation upregulates the serine pathway with increased expression of phosphoglycerate dehydrogenase (PHGDH) and phosphoserine aminotransferase (PSAT) [23]. However, it is unclear whether serine affects one-carbon metabolism to confer resistance to glutamine starvation.

Here, we performed integrated analyses of GBM cell lines, patient-derived tumorspheres, and clinical samples to examine the importance of one-carbon metabolism in response to glutamine deprivation. We demonstrated that serine and glycine were strongly present at central areas with low glutamine levels in a large number of clinical GBM samples. Glutamine starvation affected serine utilization to drive one-carbon metabolism, with increased expression of PSAT1, SHMT2, and MTHFD2. Interestingly, MTHFD2 was highly expressed in tumor samples compared with normal brain tissues, suggesting an attractive target for therapeutic intervention. Suppression of MTHFD2 expression with RNA interference promoted cell growth inhibition and death of GBM cells upon glutamine deprivation. Synthesis of serine, which is a major donor of one-carbon metabolism, was mainly mediated through autophagy rather than glycolysis. Autophagy inhibition also induced a dramatic suppression of cell proliferation and survival to GBM cells following glutamine deprivation. These results demonstrate that glutamine starvation is sufficient to change the metabolic characteristics of GBM cells and point to a previously unrecognized function of serine and one-carbon metabolism in promoting acquired adaptation to low glutamine microenvironment, indicating a new therapeutic strategy and targets for GBM patients.

## Materials and methods

Detailed protocols are found in the Additional File 1: Supplemental Experimental Procedures section.

### Cell lines

U87 and U87-EGFRvIII isogenic GBM cell lines, obtained as described previously [38], T98, and A172 human GBM cell lines (American type culture collection, ATCC) were cultured in Dulbecco’s modified Eagle’s medium (DMEM, Nacalai Tesque, Japan) supplemented with 10% FBS (Biological Industries) and 100 U/mL penicillin and streptomycin (Nacalai Tesque) in a humidified 5% CO_2_ incubator at 37 °C. For the experiments requiring medium adaptation, Dulbecco’s modified Eagle’s medium (DMEM) powder (Sigma) was used to generate media containing glucose (25 mM) and/or glutamine (4 mM). Custom-made DMEM without serine, glycine, or methionine (IFP, Japan) was used for the flux metabolite analysis. Dialyzed serum (10%) was used in these experiments.

### Patient-derived sphere cells

Human patient derived high grade glioma cells were isolated from dissociated surgical tumor specimens with approval of Kobe University Hospital Institutional Review Board [37]. Human patient derived cells were cultured in EF20 medium composed of Neurobasal medium (ThermoFisher Gibco) supplemented with 3 mM l-Glutamine (Corning), 1 × B27 supplement (ThermoFisher Gibco), 0.5 × N2 supplement (ThermoFisher Gibco), 20 ng/ml recombinant human epidermal growth factor (R&D Systems), 20 ng/ml recombinant human fibroblast growth factor-2 (PeproTech), and 0.5 × penicillin G/streptomycin/amphotericin B complex (Corning) at 37 °C and 5% CO_2_.

### Antibodies and reagents

Antibodies obtained were directed against the following: EGFR (Cell Signaling, #2232), p-EGFR Tyr1068 (Cell Signaling, #2236); EGFR/EGFRvIII cocktail antibody (Novocastra); PARP (Cell Signaling, #9542), cleaved PARP (Cell Signaling #5625); PSAT1 (Abcam ab154055); SHMT1 (Cell Signaling, #80715); SHMT2 (Cell Signaling, #12762); MTHFD1 (Abcam ab70203); MTHFD2 (Abcam ab151447); MTHFD2 (Abcam ab56772); LC3A (Cell Signaling, #4599); LC3B (Cell Signaling, #3868); and β-actin (Ambion). Reagents used are Chloroquine (CQ, Sigma) and Nicotinamide Adenine Dinucleotide reduced form (NADH, Nacalai Tesque).

### RNA extraction and real-time PCR

Total RNA from cell lines, tumor samples and normal brain tissues was extracted using a mirVana™ miRNA Isolation Kit (Applied Biosystems). First-strand cDNA was synthesized from 20 ng of total RNA using a High Capacity cDNA Reverse Transcription Kit (Applied Biosystems). Real-time RT-PCR was performed with 3 μL of diluted cDNA using TaqMan^®^ Gene Expression Assays (Applied Biosystems) following the manufacturer’s instructions. All reactions were performed in triplicate. 18S ribosomal RNA was used as the endogenous control. Quantitative mRNA expression data were acquired and analyzed by the ΔΔ-Ct method using an Applied Biosystems 7500 real-time PCR system (Applied Biosystems). TaqMan^®^ Gene Expression Assay: PSAT1(FAM): SMID: Hs00795278_mH, MTHFD1(FAM): SMID: Hs01068263_m1, MTHFD1L(FAM): SMID: Hs00914916_m1, MTHFD2(FAM): SMID: Hs00759197_s1, SHMT1(FAM): SMID: Hs00541043_g1, SHMT2(FAM): SMID: Hs01059263_g1, ATF4(FAM): SMID: Hs00909569_g1, 18S(FAM): SMID: Hs99999901_s1.

### siRNA transfection

Transfection of small interfering RNAs (siRNA) into GBM cell lines was carried out using Lipofectamine RNAiMAX (Invitrogen) in full serum, with a medium change after 24 h. Silencer® Select (Thermo Scientific, Ambion Division) specifically targeting MTHFD2 #1 (catalog 4392420, ID 21210), MTHFD2 #2 (catalog 4392420, ID 21212) and nontargeting control (LacZ) siRNAs were used at 10 nM.

### Immunohistochemical staining and image analysis-based scoring

Paraffin-embedded tissue slides were obtained from the Pathology Histology and Tissue Core Facility in Kobe University Hospital. Slides were counterstained with hematoxylin to visualize nuclei. Staining intensities were scored independently by two neuro-oncologists (KT and TS). Quantitative image analysis was performed with ImageJ software (National Institutes of Health, Bethesda, Maryland, USA) [30].

### Fluorescence microscopy

After U87 GBM cells were plated onto coverslips in 6-well plates, the growth medium was changed to the indicated treatment medium with CellLight Mitochondria-RFP reagent, BacMam 2.0 (Life Technologies), followed by a 16-h incubation at 37 °C. Cells were fixed in 4% paraformaldehyde for 20 min. Cells were permeabilized with 0.1% Triton X-100 for 30 min and blocked in 5 mg/mL bovine serum albumin (BSA) (Sigma) for 30 min. Incubation with primary antibody was performed for 1 h at room temperature followed by incubation with secondary antibody for 1 h. The following antibodies diluted in 5 mg/mL BSA, 0.1% Tween-20 in PBS were used: rabbit polyclonal MTHFD2 antibody (Abcam, ab151447, 1:100) and anti-rabbit Alexa Flour 488 IgG (H and L) antibody (Abcom, ab150073, 1:500). Coverslips were mounted with ProLong Gold Antifade Reagent and stained with 4′,6-diamidino-2-phenylindole (DAPI, Life Technologies). Images were acquired on a Keyence BZ-X700 fluorescence microscope.

### Transmission electron microscopy

Glioma cells were fixed and processed for transmission electron microscopy. Treated cells were fixed with 2.5% glutaraldehyde at 4 °C. Fixed cells were centrifugal separation at 1500 rpm 5 min, washed, and postfixed in 1% osmium tetroxide and and harden the 1%agar. After dehydration in anhydrous alcohol and propylen oxid, the cells were embedded in epoxy resin. After polymerization of the resin, ultrathin sections of the cells (60 nm) were cut on an ultramicrotome and stained with uranyl acetate and Sato’s lead staining solution. The sections were examined and photographed on a transmission electron microscope (JEM-1230; Japan Electron Optics Laboratory).

### Reactive oxygen species (ROS) analysis

Glioma cells were prepared on 6-well plates at 37 °C. 50 μM *N*-acetyl cysteine (an antioxidant) was added to some of the control and treated cells. The cells were then stained with 5 μM CellROX Green Reagent (Life Technologies) and incubated at 37 °C for 30 min. After the cells were washed with PBS, they were imaged on a Keyence BZ-X700 fluorescence microscope and analyzed with an analysis software.

### Gas chromatography mass spectrometry (GC–MS) analysis

Metabolites from biological samples were extracted and derivatized according to the method described in previous reports [20, 32]. For stable isotope-based metabolite tracing experiments, the metabolites were extracted after 24 h of incubation with [U-^13^C]-glucose (Cambridge Isotope Laboratories), not containing non-labelled glucose in all medium. Lyophilized samples were dissolved in 30 µL of dimethylformamide (Wako) and derivatized by the addition of 30 µL of *N*-tert-butyldimethylsilyl-*N*-methyltrifluoroacetamide (MTBSTFA) plus 1% tert-butylmethylchlorosilane (TMCS) (Cerilliant) at 85 °C for 60 min. The GC–MS analysis was performed using a GC–MSQP2010 Ultra (Shimadzu Co., Kyoto, Japan) with a fused silica capillary column (CP-SIL 8 CB low bleed/MS; 30 m × 0.25 mm inner diameter, 0.25 μm film thickness; Agilent Co., Palo Alto, CA). The resulting data were exported in CSV-format files and analyzed using in-house analytical software (AI output) with the in-house metabolites library [35]. For natural isotope correction, IsoCor software was used [18]. All data were normalized to the peak height of sinapinic acid (internal standard). To assess technical variation in the metabolomics experiments, each sample was extracted, derivatized, and measured in triplicate.

### Magnetic resonance spectroscopy (MRS) studies

GBM patients with newly diagnosed or recurrent gliomas underwent preoperative MRI and MRS. A 3T MRI/MRS scanner (Achieva; Philips Medical Systems, Best, The Netherlands) was used to acquire the MR spectral data, as described previously [19, 33]. Concentration estimates in absolute units of mM/L VOI were obtained with a user-independent fitting routine (LCModel; Steven Provencher, Oakville, Ontario, Canada), which is based on a library of model spectra of individual metabolites [25]. Quantification was obtained for levels of choline (Cho, 3.22 ppm), *N*-acetyl-l-aspartate (NAA, 2.0 ppm), glucose (Glc, 3.44 ppm), lactate (Lac, 1.33 ppm), glutamine (Gln, 2.45 ppm) and glutamate (Glu, 2.35 ppm). Metabolite ratios were also calculated with respect to the total creatine (Cr + phospho-Cr, 3.0 ppm), as described in previous reports [19, 33].

### Statistical analysis

The results are shown as the mean ± standard errors of the mean (SEM). Tukey–Kramer honest significance testing was performed for multiple comparison testing. Other comparisons were performed with two tailed Student’s *t* test, unless otherwise noted. Statistical significance was indicated as *p < 0.05 and **p < 0.01.

### Study approval

Glioma tissues were obtained from therapeutic procedures performed as routine clinical management at the Department of Neurosurgery, Kobe University. Tissue samples and peripheral brain tissues were resected during surgery and immediately frozen in liquid nitrogen for subsequent investigation. Each patient or their legal guardian provided written informed consent to use all clinical data and resected tissue specimens for research purposes. This study was approved by the Ethics Committee at Kobe University (approved number: 1497 for GC–MS and MRS studies of glioma patients; 1579 for use of glioma samples).

## Results

### Serine and one-carbon metabolism in GBM patients in situ

**Fig. 1:**
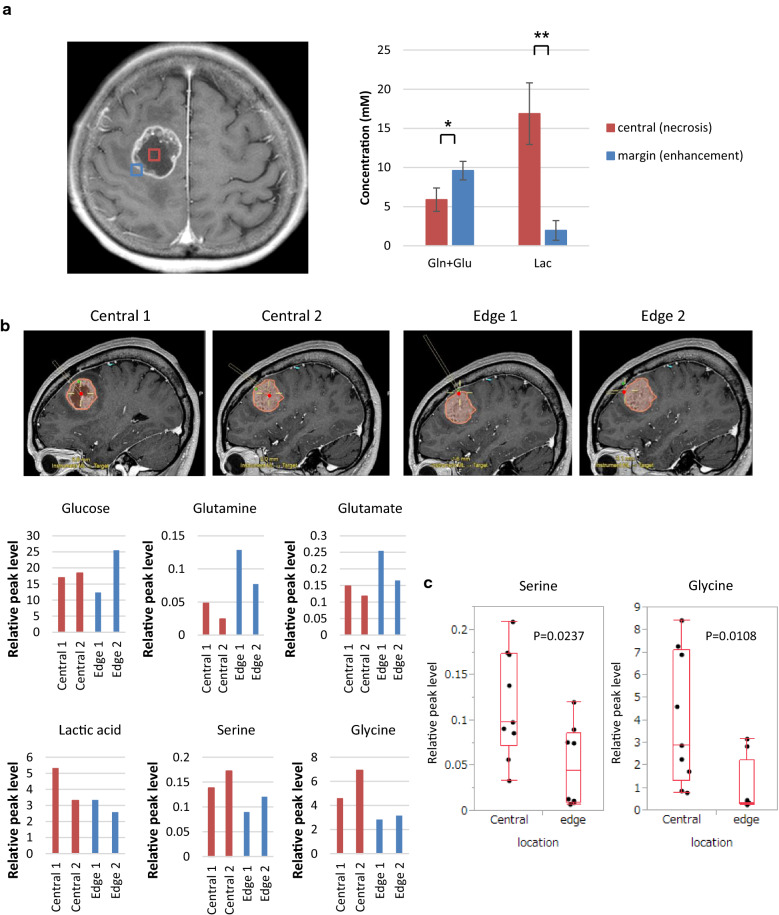
Serine and glycine levels are elevated in the core of tumor with nutrient-poor microenvironment in GBM patients. See also Additional File 2: Supplemental Fig. 1. **a** Representative MR image for MR spectroscopy (MRS) in a 68-year-old patient with GBM. Volumes of interest (VOIs) of MRS study were placed on the tumor bulk (red square) and the tumor edge (blue square). The relative level of glutamine and glutamate (Gln and Glu), and lactate (Lac) was calculated with respect to creatine and phosphocreatine (Cr and PCr) in 7 GBM patients (statistically significant with *p < 0.05, **p < 0.01). **b** GC–MS analysis in tumor samples obtained from a 60-year-old patient with GBM. Each sample was targeted in representative MR images using BrainLab navigation system. **c** Ex vivo serine and glycine levels in four GBM patients (central; 9 samples, edge; 8 samples)

Most cancer cells use two principal nutrients, glucose and glutamine to support survival and biosynthesis. Aerobic glycolysis, also known as the Warburg effect, and glutaminolysis are hallmarks of cancer cells. However, nutrients and oxygen are not always abundant within the tumor. To withstand the nutrient-limiting environments of the tumor, cancer cells must optimize nutrient utilization and alter regional metabolic activities. To explore the gradients of nutrient availability in GBM, we examined glucose and glutamine metabolism in tumor tissues (central and marginal regions of tumor) and adjacent normal brain tissues from several GBM patients. Magnetic Resonance Spectroscopy (MRS) of a 68-year-old man presenting with GBM in the right frontal lobe showed significantly higher choline and lower N-acetyl-L-aspartate (NAA) peaks in tumors than those in the contralateral normal brain (Additional File 2: Supplemental Fig. 1a). A decrease of the NAA/choline ratio is a common marker predicting increased malignancy in gliomas [24]. However, the most important and essential changes observed in this study were decreased glucose, glutamine and glutamate levels in the central region of tumors compared to the marginal tumor region in the MRS (Additional File 2: Supplemental Fig. 1a). As detected by subsequent pairwise comparisons in 7 GBM patients, glutamine and glutamate levels were significantly decreased in the center of the tumor relative to the marginal tumor region, suggesting that limiting levels of these nutrients are strongly involved in metabolic reprogramming in GBM cells (Fig. 1a). Next, stereotactic navigation-guided sampling was performed at the exact target of the tumor center and edge. Metabolites, including serine and glycine, in each sample were quantified by GC–MS. In a 60-year-old patient with GBM, four samples (two of the tumor center and two of the tumor edge) were obtained during surgery and analyzed with GC–MS (Fig. 1b). The levels of glucose, glutamine and glutamate were lower and lactate level was higher in the tumor center than in the tumor edge, which was consistent with the results of the MRS data. Interestingly, the tumor center demonstrated a higher level of serine and glycine than the tumor edge, which was consistent with the other three GBM cases (Additional File 2: Supplemental Fig. 1b). In an analysis of 17 biopsy samples of four GBM patients, serine and glycine levels were significantly higher in the tumor center than the tumor edge (Fig. 1c). Taken together, these results highlight the role of serine and glycine in tumor areas with low glutamine and glutamate levels.

### Intracellular serine and glycine levels rise in GBM cells in response to glutamine starvation

**Fig. 2:**
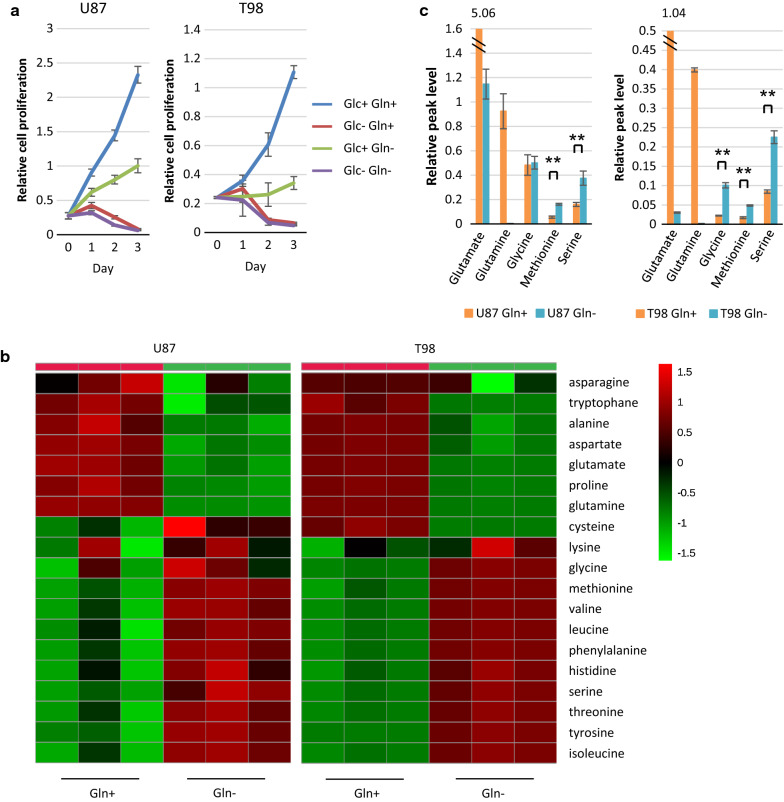
Compensatory elevation of serine and glycine levels enables GBM cells to survive glutamine starvation. See also Additional File 2: Supplemental Fig. 2. **a** U87 and T98 GBM cells were grown in the presence or absence of glucose (Glc) and/or glutamine (Gln). Cell numbers were counted over time. Data represent the mean ± SEM of three independent experiments. **b** Heatmap representation of a two-dimensional hierarchical clustering of amino acids identified as differentially expressed among U87 and T98 GBM cells which were grown with or without glutamine for 48 h. Each column represents a treatment group per cell line and each row represents an amino acid. **c** Intracellular levels of glutamine, glutamate, glycine, methionine, and serine in U87 and T98 GBM cells which were grown with and without glutamine for 48 h. Data represent the mean ± SEM of three independent experiments (statistically significant with **p < 0.01)

For most cancer cells in culture, glucose and glutamine are catabolized in appreciable quantities, supplying carbon, nitrogen, and free energy and reducing equivalents necessary to support cell growth and division [36, 39]. To explore the role of glucose and glutamine in glioma cell growth, we cultured three different glioma cell lines (U87, T98 and A172 cells) in normal medium deprived or not of glucose and/or glutamine. We found that each cell line was dependent on glucose and glutamine to support cell growth. In the absence of glucose, whether or not glutamine was present, all U87, T98 and A172 GBM cell lines failed to grow and survive, while a reduction in only glutamine availability inhibited glioma cell growth but not survival (Fig. 2a) (Additional File 2: Supplemental Fig. 2a). Because compensatory metabolic reprogramming ensured GBM cell survival under the limitation of glutamine relative to glucose, we mainly examined intracellular metabolic changes after glutamine starvation for further functional analysis. Using gas chromatography-mass spectrometry (GC-MS) of U87 and T98 GBM cells cultured in normal medium deprived or not of glutamine for 48 h, we identified 91 metabolites, including 19 amino acids, whose levels significantly changed in response to glutamine starvation (Fig. 2b) (Additional File 1: Supplemental Table 1). The heat map of variation in the metabolites for each treatment group demonstrated distinct clustering or a clear separation of each group. The key differentiating amino acids that increased after glutamine starvation were serine, glycine, and methionine, raising the possibility of efficient drive of one-carbon metabolism (Fig. 2c). We also used U87 cells overexpressing the EGFR activating mutation (U87/EGFRvIII), which is the most common EGFR mutation in GBM and increases the uptake and utilization of glucose and glutamine [33]. These results were similar after glutamine starvation in U87/EGFRvIII cells (Additional File 2: Supplemental Fig. 2b and c). Next, we determined whether hypoxic condition also influenced these metabolites, because the tumor core cells are well known to be exposed to both hypoxia and nutrient starvation, and hypoxic microenvironment plays a critical role in tumor progression and metastasis [29]. However, there was no difference in the levels of serine, glycine, and methionine between the hypoxic and normoxic conditions of U87 and T98 GBM cells (Additional File 2: Supplemental Fig. 2d). We mainly investigated the mechanism underlying glutamine starvation for further functional analysis.

### Increased MTHFD2 levels are critical for survival from glutamine starvation in GBM cells

**Fig. 3:**
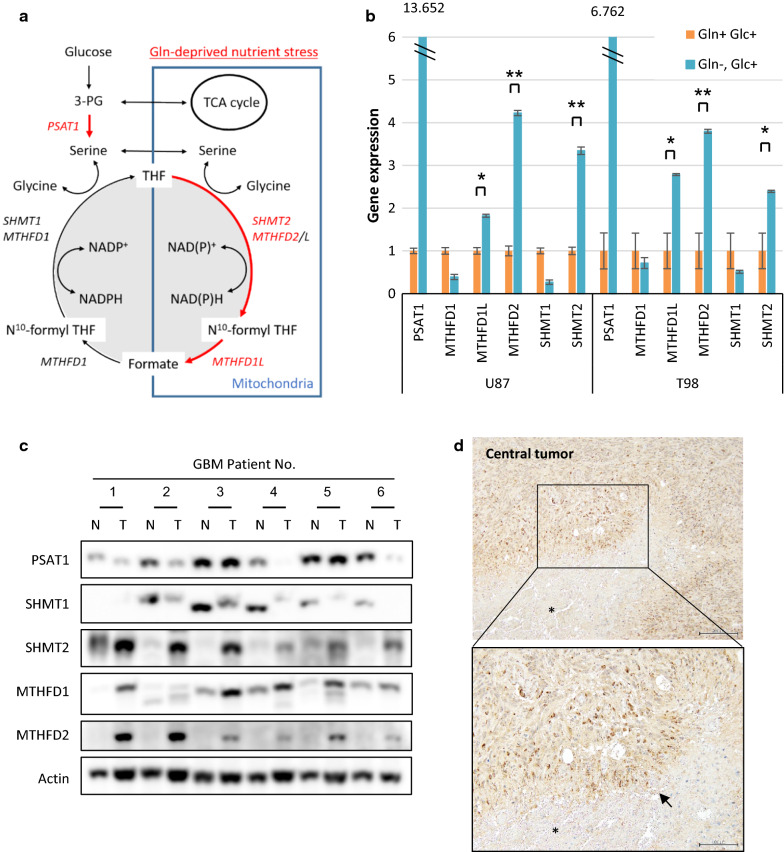
MTHFD2 expressions are elevated in the glutamine-deprived cells and tumor core of GBM patients. See also Additional File 2: Supplemental Fig. 3. **a** A schematic showing the enzymes involved in one-carbon metabolism that were targeted in this study. PSAT1; phosphoserine aminotransferase 1, SHMT1 and 2; serine hydroxymethyl transferase 1 and 2, and MTHFD1 and 2; methylenetetrahydrofolate dehydrogenase 1 and 2. MTHFD1L; monofunctional tetrahydrofolate synthase, mitochondrial **b** mRNA levels of PSAT1, SHMT1 and 2, MTHFD1 and 2, and MTHFD1L in U87 and T98 GBM cells which were grown with or without glutamine for 48 h. Data represent the mean ± SEM of three independent experiments (statistically significant with *p < 0.05, **p < 0.01). **c** Immunoblot analysis of PSAT1, SHMT1 and 2, MTHFD1 and 2 staining in central tumors (T) and normal brain tissues (N) around tumor edge obtained at tumor resection from 6 patients with GBM. **d** Representative immunohistochemical images of MTHFD2 in central tumors obtained from a GBM patient. Tissue was counterstained with hematoxylin. Scale bar upper 200 μm, lower 100 μm arrow; pseudopalisading asterisk; necrosis

Next, to identify how glutamine starvation affects one-carbon metabolism, we cultured U87 and T98 cells in normal medium deprived or not of glutamine to measure the gene expression of key enzymes in the one-carbon metabolic pathway (Fig. 3a). Notably, glutamine-deprived treatment of U87 and T98 GBM cells resulted in the upregulation of phosphoserine aminotransferase 1 (PSAT1), serine hydroxymethyl transferase 2 (SHMT2), methylenetetrahydrofolate dehydrogenase 2 (MTHFD2), and methylenetetrahydrofolate dehydrogenase 1L (MTHFD1L) (Fig. 3b), suggesting a potential metabolic flux from serine to glycine for the high rates of one-carbon metabolism in the mitochondrion. In contrast, these metabolic enzyme genes were not changed in response to glucose deprivation (Additional File 2: Supplemental Fig. 3a). Next, we extended this work by specifically focusing GBM patient-derived sphere cells (Additional File 2: Supplemental Fig. 3b). An analysis of metabolic gene levels after glutamine starvation also revealed that SHMT2, MTHFD2 and MTHFD1L mRNA expressions significantly increased in sphere-forming culture (Additional File 2: Supplemental Fig. 3c). Immunoblot analysis of lysates obtained from surgical samples of 6 GBM patients confirmed increase of MTHFD2 and SHMT2 expression in tumor tissues relative to normal brain tissues (Fig. 3c). An increase of PSAT1 levels after glutamine starvation in glioma cell lines was not observed in GBM patient-derived sphere cells and tumor core samples, meaning other factors may influence the PSAT1 signaling. In a previous paper, SHMT2 was shown to be highly expressed in the hypoxic area of GBM and provide a survival advantage to glioma cells adapting to an ischemic microenvironment [13]. Interestingly, the highest levels of MTHFD2 expression were also found in tumor cells surrounding necrotic and acellular regions, highlighting cells of what is referred to as the pseudopalisading necrosis (Fig. 3d). This dense layer of ‘‘pseudopalisading’’ viable cells is observed around necrotic tumor regions in almost all GBMs and is thought to form with limiting nutrition due to the collapse or occlusion of an intratumoral vessel [28]. Taken together, these findings suggested that one-carbon metabolism sustains GBM cell viability under low glutamine conditions and that MTHFD2 may be a good target of metabolic genes for GBM treatment.

### Genetic depletion of MTHFD2 inhibits the cell growth and survival of GBM cells with glutamine deprivation

**Fig. 4:**
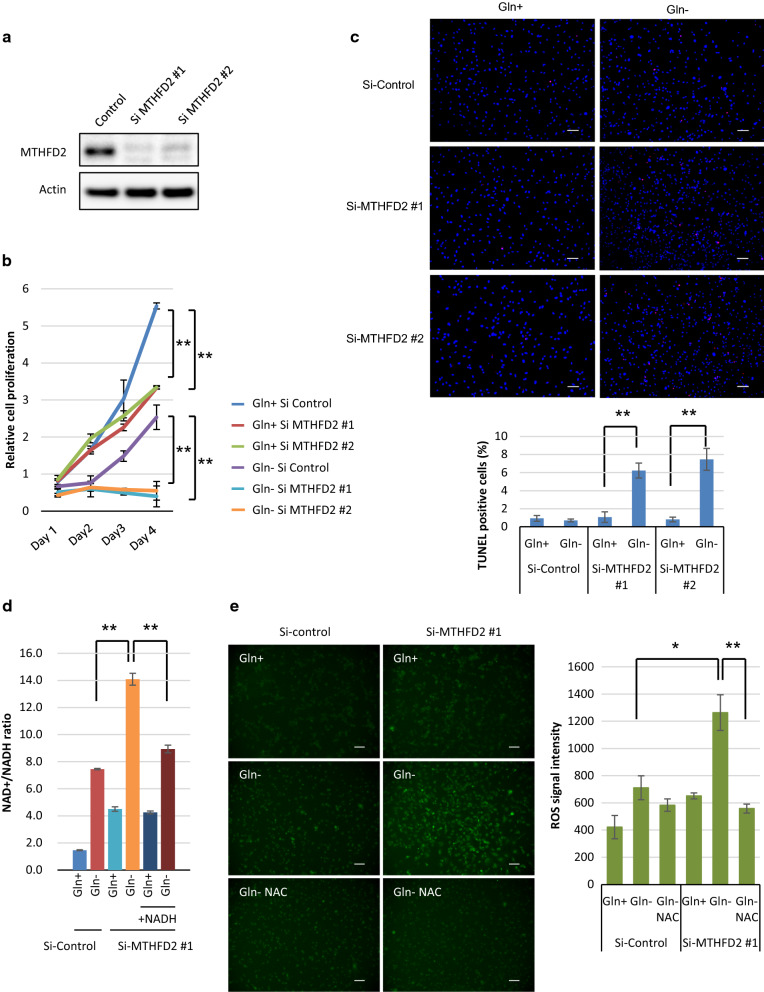
MTHFD2 expression provides survival advantage to GBM cells from glutamine deprivation. See also Additional File 2: Supplemental Fig. 4. **a**, **b** U87 cells were transfected with two types of MTHFD2 siRNA and scrambled control siRNA constructs for 24 h and changed to medium with or without glutamine at Day 1. Cell number over time represents the mean ± SEM of three independent experiments (statistically significant with **p < 0.01). Immunoblot images of MTHFD2 and actin were obtained from cell lysate. **c** Representative images of U87 cells with TUNEL staining. Scale bar: 100 μm. Cells were transfected with siRNA constructs against MTHFD2 and control LacZ which were grown with or without glutamine for 48 h. Quantification of TUNEL-positive cells was performed with the ImageJ analysis. Data represent the mean ± SEM of three independent images for each group (statistically significant with **p < 0.01). **d** NAD +/NADH ratio in U87 cells transfected with siRNA constructs against MTHFD2 and control LacZ which were grown with ± glutamine and ± NADH 1 mM for 48 h. Data represent the mean ± SEM of three independent experiments (statistically significant with **p < 0.01). **e** Representative fluorescence microscopy images of ROS signal in U87 cells. Scale bar: 100 μm. ROS measurement in U87 cells transfected with siRNA constructs against MTHFD2 and control LacZ which were grown with ± glutamine for 48 h. ROS signal was inhibited by an antioxidant, 50 mM *N*-acetyl cysteine (NAC). Data represent the mean ± SEM of three independent experiments (statistically significant with *p < 0.05, **p < 0.01)

To confirm a specific role for MTHFD2 in glutamine starvation for GBMs, we induced small interfering RNA (siRNA)-mediated MTHFD2 knockdown in U87 GBM cells and assessed its impact on cell death in response to glutamine starvation. MTHFD2 knockdown was confirmed using two types of siRNA construction in U87 GBM cells (Fig. 4a). Genetic depletion of MTHFD2 by siRNA transfection inhibited the proliferation of all GBM cells, with enhanced anti-proliferative effects upon glutamine starvation (Fig. 4b). Of note, knockdown of MTHFD2 effectively sensitized U87 cells to glutamine starvation-mediated cell death, as indicated by TUNEL-positive cells (Fig. 4c). Serine-derived one-carbon units are used in the folate cycle, which is essential for nucleotide synthesis and the generation of NAPDH, NADH and ATP. For nucleotide synthesis, we first quantified metabolites related to purine and pyrimidine metabolism by liquid chromatography-mass spectrometry (LC–MS) after glutamine starvation, but no suggestive change was found in U87 or T98 GBM cells (Additional File 2: Supplemental Fig. 4a). The MTHFD1-dependent reduction of 10-formyl-THF to 5,10-methylene-THF is energetically facilitated by a higher NADPH/NADP + ratio in the cytosol, whereas the MTHFD2 reaction is driven by a more oxidative mitochondrial redox potential that favors the use of NAD + by MTHFD2 [41]. To confirm the role of MTHFD2 in redox homeostasis, we analyzed the intracellular NAD +/NADH ratio. Glutamine starvation resulted in a high NAD +/NADH ratio in U87 GBM cells for redox maintenance. Importantly, knockdown of MTHFD2 showed an increase in the NAD +/NADH ratio, which was rescued by providing NADH in the culture medium (Fig. 4d). This indicates that MTHFD2 depletion inhibited NAD + application for redox maintenance, leading to extensive cell death upon glutamine starvation. To assess the direct link to increased cytotoxicity via redox status, we have performed the reactive oxygen species (ROS) assays in U87 and T98 GBM cells. Glutamine-deprived ROS signal increased by MTHFD2 knockdown, which was inhibited by an antioxidant (Fig. 4e) (Additional File 2: Supplemental Fig. 4b). Taken together, these findings suggest that serine-mediated one-carbon metabolism is also key for the metabolic alteration of GBM, especially in the tumor microenvironment of low glutamine, and demonstrate a previously unknown role for MTHFD2 in mediating glutamine-deprived stress by redox maintenance through one-carbon metabolism in GBM cells.

### GBM cells use autophagy-mediated recycling of serine to survive glutamine deprivation

**Fig. 5:**
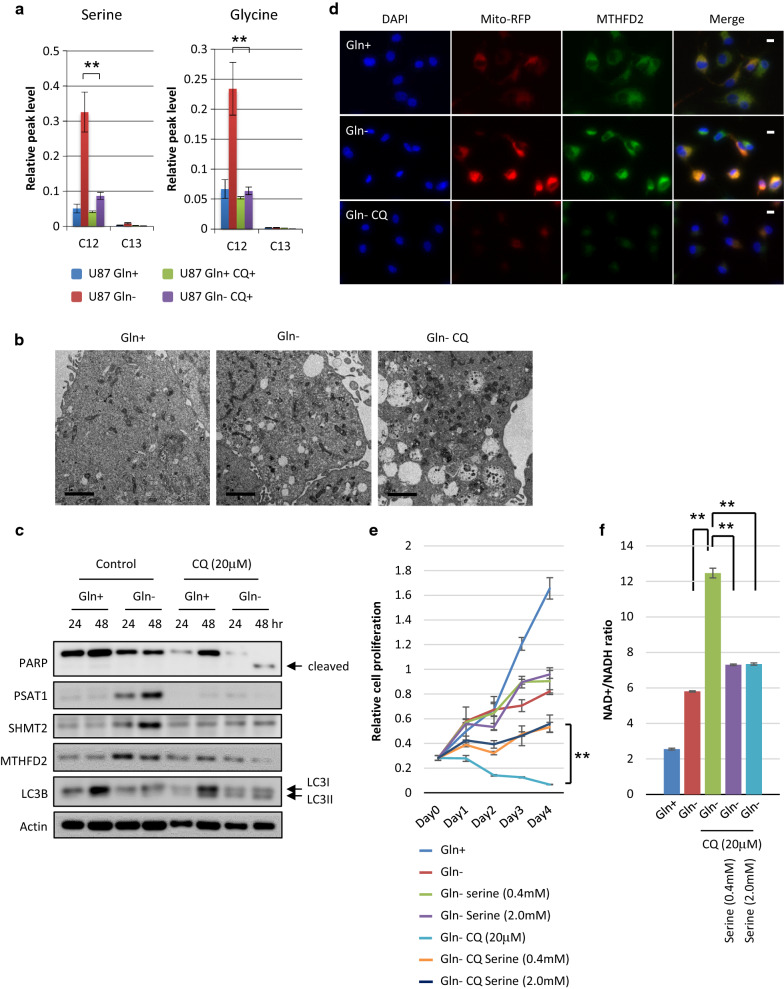
Serine is supplied by autophagy of GBM cells to survive glutamine deprivation. See also Additional File 2: Supplemental Fig. 5. **a** GC–MS analysis of glutamine (Gln) starvation and 20 μM chloroquine (CQ) treatment effect on [U-^13^C] glucose metabolism to different ^13^C isotopologues of serine and glycine in U87 GBM cells treated for 24 h. Data represent the mean ± SEM of three independent experiments (statistically significant with **p < 0.01). **b** Representative transmission electron microscopy images of U87 GBM cells which were grown with or without glutamine (Gln) and treated with chloroquine (CQ) for 24 h. Scale bar: 2 μm **c** Immunoblot analysis using indicated antibodies of U87 cells which were grown with or without glutamine and treated with chloroquine (CQ) or control DW for 24–48 h. **d** U87 GBM cells in glutamine-starved and treated with 20 μM chloroquine (CQ) for 24 h. Cells were then fixed, permeabilized and stained for DNA (DAPI), mitochondria (Mito-RFP) and MTHFD2. One representative picture for each condition is shown. Scale bar: 10 μm **e** U87 GBM cells were grown without glutamine (Gln) and treated with 20 μM chloroquine (CQ) in the presence or absence of 0.4 and 2.0 mM serine. Cell numbers were counted over time. Data represent the mean ± SEM of three independent experiments (statistically significant with **p < 0.01). **f** NAD +/NADH ratio in U87 cells which were grown with or without glutamine (Gln) treated with 20 μM chloroquine (CQ) or DW in medium containing 0.4 and 2.0 mM serine or not for 48 h. Data represent the mean ± SEM of three independent experiments (statistically significant with **p < 0.01)

Although serine can be taken up into the cell using a number of different transporters or can be synthesized by the cell, glucose is the major source of carbons for de novo serine synthesis in cancer cells. We sought to determine whether glucose entry into de novo serine synthesis would be compromised by glutamine starvation. U87 glioma cells were cultured in serine, glycine-free medium deprived or not of glutamine and analyzed by GC–MS to quantify glucose-derived 13C isotopologues of metabolites. 13C-labeled lactate and glutamate levels decreased in response to glutamine starvation, suggesting that glycolysis and glutamine-dependent anaplerosis might be not promoted under glutamine limitation (Additional File 2: Supplemental Fig. 5a). Importantly, compared with level of 13C-labeled serine and glycine and methionine, 12C-labeled levels dramatically increased (Fig. 5a) (Additional File 2: Supplemental Fig. 5a), meaning that glucose-derived serine synthesis was not accelerated under glutamine-deprived conditions. Next, to assess the possibility that autophagy could be essential to sustain metabolism, energy homeostasis, and survival upon glutamine starvation, we tested the effect of the autophagy inhibitor chloroquine on mediating the cellular response to glutamine starvation. Strikingly, increased levels of 12C-labeled serine, glycine and methionine were inhibited by chloroquine treatment (Fig. 5a) (Additional File 2: Supplemental Fig. 5a), suggesting that serine synthesis is mediated through autophagy rather than glycolysis. In transmission electron microscopy images, few autophagic vesicles (AVs) were found within control cells cultured in full nutrients, but were abundant in glutamine-starved cells. Of note, glutamine-starved cells treated with 20 mM chloroquine showed many large AVs that contained undigested organelles, confirming that glutamine starvation induces autophagy in glioma cells (Fig. 5b). LC3 fluorescence staining also indicated that chloroquine significantly enhanced LC3 expression of GBM cells following glutamine starvation (Additional File 2: Supplemental Fig. 5b). Chloroquine induced significant cell death of GBM cells upon glutamine starvation, as indicated by cleaved PARP, and suppressed the upregulated levels of metabolic genes, such as PSAT1, SHMT2, and MTHFD2 (Fig. 5c). To study the glutamine starvation and chloroquine treatment response of MTHFD2 protein in GBM cells with better time resolution, we monitored MTHFD2 expression in U87 GBM single cells using fluorescence microscopy. Consistent with the immunoblot results (Fig. 5c), MTHFD2 protein levels in the mitochondria in single cells increased markedly when deprived of glutamine (Fig. 5d). Upon chloroquine treatment, MTHFD2 expression was significantly reduced (Fig. 5d). Chloroquine also suppressed the glutamine-deprived GBM cell proliferation in a dose-dependent manner (Additional File 2: Supplemental Fig. 5c). Importantly, serine rescued the viability of glutamine-deprived U87 GBM cells treated with the autophagy inhibitor (Fig. 5e). In GBM cells starved of glutamine, chloroquine significantly enhanced the NAD +/NADH ratio, which was rescued by replenishment of serine (Fig. 5f). Taken together, these findings raise the possibility that autophagy-dependent serine supply into glioma cells is strongly involved in driving one-carbon metabolism to enable GBM cells to survive glutamine starvation. These results also suggest that autophagy inhibition is a reasonable therapeutic strategy to target glioma cell adaptation to the tumor microenvironment.

## Discussion

Cancer cells exhibit metabolic alterations of oncogenic and tumor suppressor pathways throughout tumor development. One metabolic feature of many cancer cells is high glycolytic flux to lactate in the presence of oxygen compared to normal cells, a phenomenon known as aerobic glycolysis or the ‘‘Warburg effect’’ [17]. Glutamine can also supply new carbon to the tricarboxylic acid (TCA) cycle (anaplerosis) in order for TCA-cycle intermediates to be removed from the cycle and used for the production of new macromolecules [6]. Although glucose and glutamine are the major nutrients in cancer cells, changes in nutrient and oxygen concentrations due to vascular dynamics during tumor development can influence the metabolic properties of tumors. It is not entirely clear how tumors deal with low nutrient and oxygen concentrations as suitable cellular responses. It is possible that other nonglutamine amino acids are used as nutrient sources, and quantitative analysis of carbon sources for biomass production suggests a significant contribution of amino acids as biosynthetic precursors for protein [11]. Aspartate, for example, modulates the NADH-fumarate reductase system to maintain mitochondrial energy production under hypoxic-hypoglycemic conditions [34]. Pancreatic stellate cell-derived alanine also acts as an alternative carbon source to fuel the tricarboxylic acid (TCA) cycle in pancreatic ductal adenocarcinoma cells [31]. Glutamine withdrawal leads to upregulation of the serine pathway in leukemia cells [23]. We have found that serine expression is significantly elevated in glutamine-deprived GBM cells, as well as in the necrotic tumor core with extremely low nutrients in GBM patients. These results suggest a potential mechanism underlying the metabolic reprogramming against some metabolic stress.

Serine is a nonessential amino acid that can be synthesized from glucose and is important for de novo synthesis of ATP, which may have a broad influence on cellular metabolism. Serine is crucial for multiple metabolic pathways required for cell growth and proliferation, including phospholipid, purine and glutathione biosynthesis, as well as being a methyl source for one-carbon metabolism [16]. As a downstream player in the folate pathway, one-carbon metabolism, including serine and glycine, is activated in many cancer cells, with high levels of the mitochondrial enzyme genes SHMT2 and MTHFD2 [15, 22]. MTHFD2 expression is markedly elevated in many cancers and correlates with poor survival in breast cancer [22]. SHMT2 has also been reported to be highly expressed in the pseudopalisading cells that surround ischemic necrotic foci in human GBM [13]. Hypoxia-inducible factors (HIF) has reported to regulate expression of genes encoding PHGDH and five downstream enzymes including MTHFD2 and SHMT2 in the serine synthesis pathway and mitochondrial one-carbon cycle in breast cancer stem cells [29]. We too have found that increased levels of both MTHFD2 and SHMT2 regulate one-carbon metabolism, allowing GBM cell lines and GBM patient-derived tumorspheres to survive low glutamine conditions. A recent study demonstrated that mTORC1 signaling in cancer cells increases metabolic flux though purine synthesis via expression of MTHFD2 [1]. Although glutamine can regulate mTORC1 signaling [21], our data may suggest a different pathway in GBM cells, which are resistant to glutamine starvation. Altered metabolism is considered to be a key role of cancer progression and survival in many cancers and provide insights into effective therapies for the treatment of GBM patients.

One-carbon metabolism supports multiple physiological processes including biosynthesis, amino acid homeostasis, epigenetic maintenance and redox defense [7]. We demonstrated that glutamine derivation activates serine synthesis and one-carbon metabolism for redox maintenance, such as reactive oxygen species (ROS), rather than energy generation and nucleotide biosynthesis in GBM cells. Although the cytosolic/nuclear and mitochondrial pools of NAD+ are distinct and interconnected by an intricate set of cellular redox processes, the enhancement of NAD+ levels has been linked with improved mitochondrial function under stress, leading to protection against dietary limitation [3]. This also highlights the potential mechanisms interconnecting mitochondrial NAD+ pools, as their homeostasis and interaction are essential for the preservation of cell survival from nutrient starvation.

Cancer cells must optimize nutrient utilization when resources are scarce [2]. Glycolytic flux is crucial for the rapid proliferation of cancer cells, and glucose incorporation can be increased to compensate for the absence of some nutrients. Cells are known to have increased dependence on glucose-derived serine when serine is limiting [4, 14]. In our study, however, serine was not derived from glucose but autophagy under glutamine limiting conditions. In starvation, autophagy is well known to be rapidly induced for recycling intracellular components to support metabolism in many cancers. Autophagy is also essential for maintenance of the functioning pool of mitochondria in cancer cells [27]. Indeed, we found that mitochondrial genes, such as SHMT2 and MTHFD2, were upregulated after glutamine starvation. Previous studies have shown that rapid and extensive mitochondrial tabulation and elongation are found during starvation-induced autophagy and sustained cell viability [10, 26]. This suggested that crosstalk between mitochondria and autophagy influences cellular survival. However, further studies will be needed to explore the more detailed roles of one-carbon metabolism in the mitochondrial system during nutrient starvation.

In summary, glutamine-deprived GBM cells showed higher levels of serine, cysteine, and methionine with upregulated gene expression of PSAT1, SHMT2, and MTHFD2, to regulate serine synthesis and one-carbon metabolism. In human glioma samples, MTHFD2 expression was highest in the nutrient-poor regions around “pseudopalisading necrosis.” Serine synthesis was mediated through autophagy rather than glycolysis. Importantly, suppression of MTHFD2 and autophagy inhibition impaired glioma cells in glutamine-deprived conditions. These findings may have important implications for serine-dependent one-carbon metabolism for glioma cells to survive glutamine starvation and suggest a new therapeutic strategy for patients with malignant glioma.

## Supplementary Information

**Additional File 1.** Supplementary figures. **Fig. S1**: Metabolomic analysis in the ‘central’ and ‘edge’ of tumor of GBM patients. **Fig. S2**: Serine and glycine levels in glioma cells treated with glutamine starvation and hypoxia. **Fig. S3**: MTHFD2 expressions after glutamine starvation in GBM patient-derived sphere cells. **Fig. S4**: Reactive oxygen species (ROS) status and nucleotide biosynthesis in glutamine-deprived GBM cells. **Fig. S5**: The role of autophagy in one-carbon metabolism of GBM cells to survive glutamine deprivation.

**Additional File 2.** Supplementary materials: Experimental Procedures and references. Supplementary figure legends: **Fig. S1-S5**. Supplementary Table 1: Metabolites identified in GC-MS analysis.
