# Supplementary material for: Glioma cells require one-carbon metabolism to survive glutamine starvation
Source: Acta Neuropathol Commun. 2021 Jan 19;9:16. doi: 10.1186/s40478-020-01114-1 (PMC7814586; doi:10.1186/s40478-020-01114-1)
Supplement: Supplementary file 1 — Additional File 1. Supplementary figures. Fig. S1: Metabolomic analysis in the ‘central’ and ‘edge’ of tumor of GBM patients. Fig. S2: Serine and glycine levels in glioma cells treated with glutamine starvation and hypoxia. Fig. S3: MTHFD2 expressions after glutamine starvation in GBM patient-derived sphere cells. Fig. S4: Reactive oxygen species (ROS) status and nucleotide biosynthesis in glutamine-deprived GBM cells. Fig. S5: The role of autophagy in one-carbon metabolism of GBM cells to survive glutamine deprivation. [file 40478_2020_1114_MOESM1_ESM.docx]

**List of Supplementary Materials:**

Supplemental Experimental Procedures

Supplemental Figure legends

Supplemental Table

**Supplemental Experimental Procedures**

**Cell proliferation and death assays**

Cells were placed in 96-well plates at 2x10^3^ cells/well in 100 μL of growth medium and then incubated for 48 hours in each treatment condition. Cell proliferation was examined with Cell Count Reagent SF (Nacalai Tesque) according to the manufacturer’s instructions. The absorbance of the treated and untreated cells was measured with a microplate reader (Thermo Scientific) at 450 nm. Cell death was assessed by trypan blue exclusion (Nacalai Tesque).

**Western blotting**

Cultured cells or snap-frozen tissue samples were lysed and homogenized with a Lysis buffer AM1 and phosphatase inhibitor and protease inhibitor cocktail (Active Motif). Equal amounts of protein extracts were separated by electrophoresis on 4-12 % NuPAGE Bis-Tris Mini Gels (Invitrogen) and then transferred to a nitrocellulose membrane (GE Healthcare) with the XCell II Blot Module (Invitrogen). The membrane was blocked for 1 hour in Tris-buffered saline containing 0.1 % Tween-20 and 5 % nonfat milk and then probed with various primary antibodies, followed by secondary antibodies conjugated to horseradish peroxidase (HRP). The immunoreactivity was revealed with Super Signal West Pico Chemiluminescent Substrate or the West Femto Trial Kit (Thermo Scientific).

**TUNEL staining**

Cells were placed in 6-well chamber slides at 1x10^5^ cells/well in 5 mL of growth medium and then incubated for 48 hours in each treatment condition. Apoptotic cells were evaluated with the In Situ Cell Death Detection Kit, Fluorescein and following the manufacturer’s protocol (Roche). Nuclei were stained blue by 4′, 6-diamidino-2-phenylindole (DAPI) (Invitrogen). TUNEL-positive cells were visualized with a fluorescein microscope (Keyence BZ-9000, Japan). The percentage of apoptosis was calculated as the percentage of TUNEL-positive cells out of 400 cells from each group using the NIH images.

**Analysis of purine and pyrimidine pathway intermediates by coupling ion chromatography and liquid chromatography methods to mass spectrometry**

Metabolite extraction was performed using the Bligh and Dyer’s method with modifications [1]. Hydrophilic metabolite extracts were analyzed by an ion chromatography with a Dionex IonPac AS11-HC-4 μm column (2 mm i.d. × 250 mm, 4 μm particle size, Thermo Fisher Scientific, Waltham, MA, USA) coupled with a quadrupole-Orbitrap mass spectrometry (IC/MS) (Thermo Fisher Scientific) for anionic polar metabolites (i.e., nucleotides) [2, 3] and a liquid chromatography with a Discovery HS F5 column (2.1 mm i.d. × 150 mm, 3 μm particle size, Merck, Darmstadt, Germany) coupled with a quadrupole-Orbitrap mass spectrometry (PFPP-LC/MS/MS) (Thermo Fisher Scientific) for cationic polar metabolites (i.e., bases and nucleosides) [3, 4].

**References**

1 Bligh EG, Dyer WJ (1959) A rapid method of total lipid extraction and purification. Can J Biochem Physiol 37: 911-917 Doi 10.1139/o59-099

2 Hu S, Wang JH, Ji EH, Christison T, Lopez L, Huang YY (2015) Targeted Metabolomic Analysis of Head and Neck Cancer Cells Using High Performance Ion Chromatography Coupled with a Q Exactive HF Mass Spectrometer. Anal Chem 87: 6371-6379 Doi 10.1021/acs.analchem.5b01350

3 Izumi Y, Matsuda F, Hirayama A, Ikeda K, Kita Y, Horie K, Saigusa D, Saito K, Sawada Y, Nakanishi Het al (2019) Inter-Laboratory Comparison of Metabolite Measurements for Metabolomics Data Integration. Metabolites 9: Doi ARTN 257 10.3390/metabo9110257

4 Yoshida H, Mizukoshi T, Hirayama K, Miyano H (2007) Comprehensive analytical method for the determination of hydrophilic metabolites by high-performance liquid chromatography and mass spectrometry. J Agr Food Chem 55: 551-560 Doi 10.1021/jf061955p

**Supplemental Figure legends**

**Supplemental Fig. 1**

**Metabolomic analysis in the ‘central’ and ‘edge’ of tumor of GBM patients. See also Fig. 1.**

(a) MR spectroscopy (MRS) studies targeting glucose, glutamine and glutamate for tumor bulk (blue), tumor edge (red) and contralateral normal brain (green) regions in a 68-year-old patient with GBM. The peak of glucose, choline, glutamine and glutamate complex, and N-acetyl-L-aspartate is around 3.44, 3.22, 2.4 and 2.0 ppm of chemical shift, respectively. Cho; choline, Cr; Creatine, Glc; glucose, Gln; glutamine, Glu; glutamate, NAA; N-acetyl-L-aspartate. (b) GC-MS analysis for serine and glycine in tumor samples obtained from three patients with GBM. Each sample was targeted in representative MR images using BrainLab navigation system.

**Supplemental Fig. 2**

**Serine and glycine levels in glioma cells treated with glutamine starvation and hypoxia. See also Fig. 2.**

(a) A172 GBM cells were grown in the presence or absence of glucose (Glc) and/or glutamine (Gln). Cell numbers were counted over time. Data represent the mean ± SEM of three independent experiments. (b) Heatmap representation of a two-dimensional hierarchical clustering of amino acids identified as differentially expressed among U87/EGFRvIII GBM cells which were grown with or without glutamine for 48 hours. Each column represents a treatment group per cell line and each row represents an amino acid. (c) Intracellular levels of glutamine, glutamate, glycine, methionine, and serine in U87/EGFRvIII GBM cells which were grown with and without glutamine for 48 hours. Data represent the mean ± SEM of three independent experiments (statistically significant with **P<0.05, **P<0.01*). (d) Intracellular levels of glycine, methionine, and serine in U87 and T98 GBM cells cultured in normoxia (O_2_ 20 %) and hypoxia (O_2_ 1.5 %) conditions for 48 hours. Data represent the mean ± SEM of three independent experiments.

**Supplemental Fig. 3**

**MTHFD2 expressions after glutamine starvation in GBM patient-derived sphere cells. See also Fig. 3.**

(a) mRNA levels of PSAT1, SHMT1 and 2, MTHFD1 and 2, and MTHFD1L in U87 and T98 GBM cells which were grown with or without glucose for 48 hours. Data represent the mean ± SEM of three independent experiments. (b) Schematic showing the experimental tumorsphere models established from a 69-year-old patient with GBM. Scale bar 200μm. (c) mRNA levels of PSAT1, SHMT1 and 2, MTHFD1 and 2, and MTHFD1L in GBM patient-derived sphere cells which were grown with or without glutamine for 48 hours. Data represent the mean ± SEM of three independent experiments (statistically significant with **P<0.05*).

**Supplemental Fig. 4**

**Reactive oxygen species (ROS) status and nucleotide biosynthesis in glutamine-deprived GBM cells. See also Fig. 4.**

(a) Heatmap representation of a two-dimensional hierarchical clustering of metabolites identified as differentially expressed among U87 and T98 GBM cells which were grown with or without glutamine for 48 hours. Each column represents treatment groups per cell line and each row represents metabolites related to purine and pyrimidine metabolism. (b) ROS measurement in T98 GBM cells transfected with siRNA constructs against MTHFD2 and control LacZ which were grown with +/-glutamine for 48 hours. ROS signal was inhibited by an antioxidant, 50 mM N-acetyl cysteine (NAC). Data represent the mean ± SEM of three independent experiments (statistically significant with **P<0.05*).

**Supplemental Fig. 5**

**The role of autophagy in one-carbon metabolism of GBM cells to survive glutamine deprivation. See also Fig. 5.**

(a) GC-MS analysis of glutamine (Gln) starvation and 20 μM chloroquine (CQ) treatment effect on [U-^13^C] glucose metabolism to different ^13^C isotopologues of lactate, glutamine, glutamate, and methionine in U87 GBM cells treated for 24 hours. Data represent the mean ± SEM of three independent experiments. (b) U87 GBM cells grown with or without glutamine (Gln) treated with 20 μM chloroquine (CQ) or DW for 48 hours were stained with LC3 and examined by fluorescence microscopy. Scale bar, 50 μm. (c) U87 and T98 GBM cells were grown without glutamine (Gln) and treated with 10-40 μM chloroquine (CQ) or DW. Cell numbers were counted over time. Data represent the mean ± SEM of three independent experiments (statistically significant with **p<0.05, **p<0.01*).

**Supplemental Table 1. Metabolites identified in GC-MS analysis, related to Fig. 2.**

| **No.** | **Metabolite Name** | |  |  |  |  |
| --- | --- | --- | --- | --- | --- | --- |
| **1** | 1,6-Anhydroglucose | **31** | | Fructose | **61** | N-Acetyl-L-Aspartic acid |
| **2** | 2,3-Bisphospho-glycerate | **32** | | Fructose-6-Phosphate | **62** | n-Caprylic acid |
| **3** | 2-Aminobutyric acid | **33** | | Fumaric acid | **63** | Nicotinamide |
| **4** | 2-Aminoethanol | **34** | | Galactitol | **64** | N-Methylethanolamine |
| **5** | 2-Aminoisobutyrate | **35** | | Galactosamine | **65** | Nonanoic acid |
| **6** | 2-Aminopimelic acid | **36** | | Galacturonic acid | **66** | Ornithine |
| **7** | 2-Dehydro-D-gluconate | **37** | | Glucose | **67** | Oxalate |
| **8** | 2'-Deoxyribose-5'-Phosphate | **38** | | Glutamic acid | **68** | Pantothenate |
| **9** | 2-Thiouracil | **39** | | Glutamine | **69** | Phenylalanine |
| **10** | 3-Hydroxyisovaleric acid | **40** | | Glycerol-2-Phosphate | **70** | Proline |
| **11** | Acetoacetic acid | **41** | | Glycine | **71** | Putrescine |
| **12** | Aconitate | **42** | | Glycyl-Glycine | **72** | Pyroglutamic acid |
| **13** | Adenine | **43** | | Heptadecanoate | **73** | Pyruvate+Oxalacetic acid |
| **14** | Adenylosuccinic acid | **44** | | Histidine | **74** | Ribitol |
| **15** | Alanine | **45** | | Homocysteine | **75** | Sarcosine |
| **16** | Allantoin | **46** | | Hypoxanthine | **76** | Serine |
| **17** | Allothreonine | **47** | | Inositol | **77** | Spermidine |
| **18** | Anthranilic acid | **48** | | Kynurenine | **78** | Succinic acid |
| **19** | Arabinose-5-phosphate | **49** | | Lactic acid | **79** | Sucrose |
| **20** | Ascorbic acid | **50** | | Lactitol | **80** | Tagatose |
| **21** | Asparagine | **51** | | Lauric acid | **81** | Tartarate |
| **22** | Aspartic acid | **52** | | Leucine | **82** | Taurine |
| **23** | b-Alanine | **53** | | Lysine | **83** | Threonine |
| **24** | Cadaverine | **54** | | Lyxose | **84** | trans-4-Hydroxy-L-proline |
| **25** | Citric acid + Isocitric acid | **55** | | Malic acid | **85** | Tryptophan |
| **26** | Citrulline | **56** | | Malonic acid | **86** | Tyrosine |
| **27** | Creatinine | **57** | | Maltotriose | **87** | Uracil |
| **28** | Cysteic acid | **58** | | meso-erythritol | **88** | Urea |
| **29** | Cysteine Sulfonic acid | **59** | | Methionine | **89** | Uridine |
| **30** | Cysteine+Cystine | **60** | | N-a-Acetyl-L-Lysine | **90** | Valine |
|  |  |  | |  | **91** | Xylitol |
