# Supplementary material for: Glioma cells require one-carbon metabolism to survive glutamine starvation
Source: Acta Neuropathol Commun. 2021 Jan 19;9:16. doi: 10.1186/s40478-020-01114-1 (PMC7814586; doi:10.1186/s40478-020-01114-1)
Supplement: Supplementary file 2 — Additional File 2. Supplementary materials: Experimental Procedures and references. Supplementary figure legends: Fig. S1-S5. Supplementary Table 1: Metabolites identified in GC-MS analysis. [file 40478_2020_1114_MOESM2_ESM.pptx]

## Slide 1
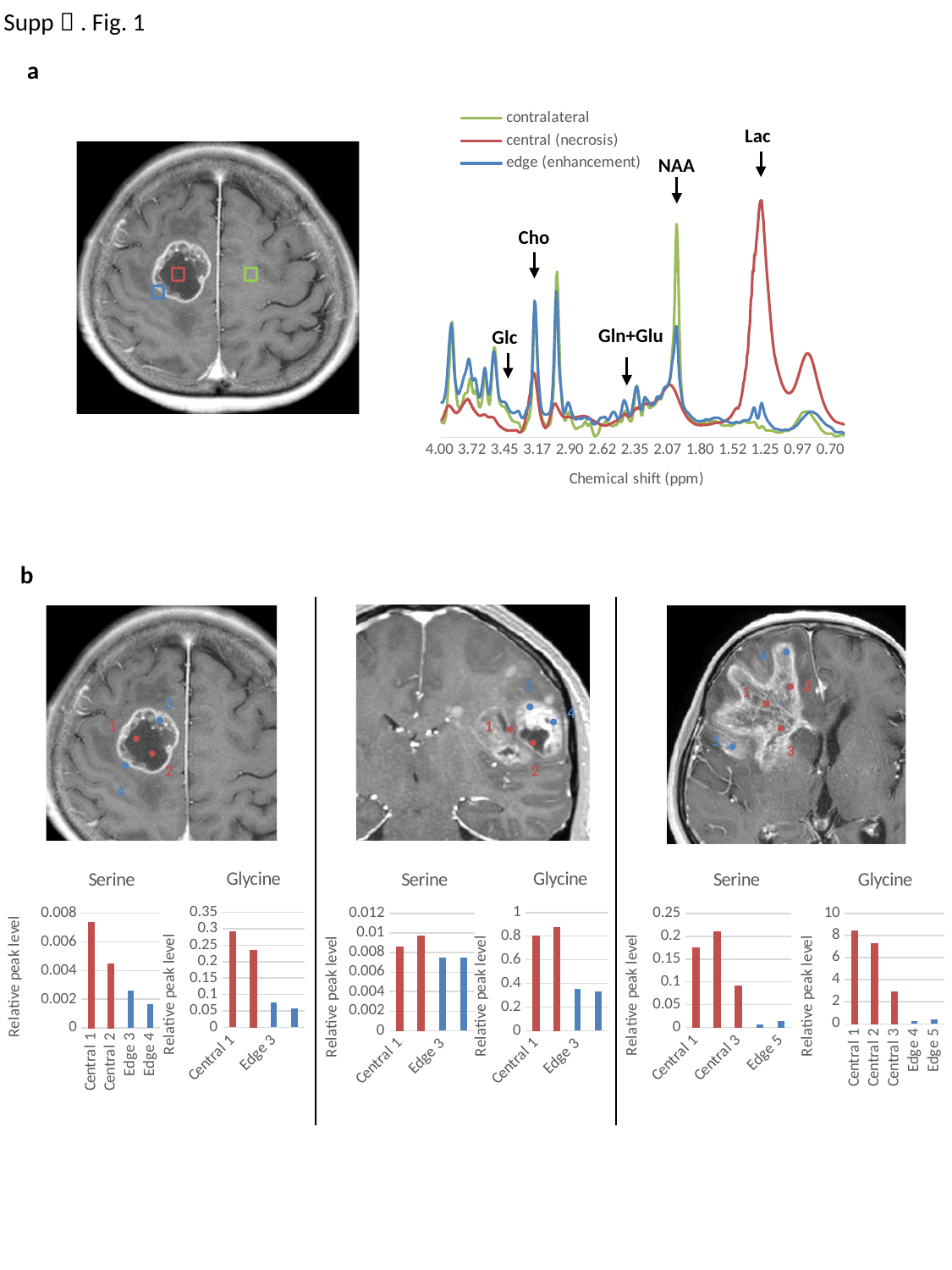

Suppｌ. Fig. 1
a
### Chart
| Category | contralateral | central (necrosis) | edge (enhancement) |
|---|---|---|---|
| 4 | 292973.0 | 314542.0 | 628623.0 |
| 3.992356 | 261682.0 | 327883.0 | 629301.0 |
| 3.9847109999999999 | 250471.0 | 368210.0 | 646962.0 |
| 3.9770669999999999 | 263236.0 | 398489.0 | 689750.0 |
| 3.9694219999999998 | 315282.0 | 461483.0 | 761926.0 |
| 3.9617779999999998 | 380070.0 | 490117.0 | 860561.0 |
| 3.9541339999999998 | 514723.0 | 544666.0 | 1031650.0 |
| 3.9464890000000001 | 776603.0 | 559442.0 | 1284200.0 |
| 3.9388450000000002 | 1065210.0 | 577171.0 | 1528300.0 |
| 3.9312 | 1376270.0 | 573530.0 | 1764660.0 |
| 3.923556 | 1746650.0 | 556533.0 | 1972740.0 |
| 3.9159109999999999 | 2051440.0 | 537257.0 | 2050530.0 |
| 3.9082669999999999 | 2087110.0 | 517463.0 | 1904810.0 |
| 3.900623 | 1853290.0 | 480995.0 | 1585550.0 |
| 3.8929779999999998 | 1490600.0 | 477725.0 | 1256260.0 |
| 3.8853339999999998 | 1133280.0 | 444448.0 | 1043420.0 |
| 3.8776890000000002 | 873552.0 | 457014.0 | 943292.0 |
| 3.8700450000000002 | 703678.0 | 438322.0 | 861855.0 |
| 3.8624010000000002 | 592715.0 | 460302.0 | 788669.0 |
| 3.8547560000000001 | 542256.0 | 458352.0 | 780815.0 |
| 3.8471120000000001 | 530212.0 | 494127.0 | 813327.0 |
| 3.839467 | 540571.0 | 504058.0 | 861302.0 |
| 3.831823 | 557487.0 | 551915.0 | 909277.0 |
| 3.8241779999999999 | 602833.0 | 562007.0 | 971908.0 |
| 3.8165339999999999 | 674511.0 | 608261.0 | 1040800.0 |
| 3.8088899999999999 | 747412.0 | 615242.0 | 1099390.0 |
| 3.8012450000000002 | 789784.0 | 646807.0 | 1141890.0 |
| 3.7936009999999998 | 797456.0 | 666825.0 | 1189730.0 |
| 3.7859560000000001 | 798032.0 | 672912.0 | 1262400.0 |
| 3.7783120000000001 | 835358.0 | 698783.0 | 1352650.0 |
| 3.7706680000000001 | 931339.0 | 663063.0 | 1417460.0 |
| 3.763023 | 1034070.0 | 669554.0 | 1398860.0 |
| 3.755379 | 1066410.0 | 606513.0 | 1288120.0 |
| 3.7477339999999999 | 1016010.0 | 600094.0 | 1150760.0 |
| 3.7400899999999999 | 903630.0 | 536720.0 | 1051480.0 |
| 3.7324449999999998 | 804886.0 | 535450.0 | 1025530.0 |
| 3.7248009999999998 | 785215.0 | 484308.0 | 1050490.0 |
| 3.7171569999999998 | 828388.0 | 484348.0 | 1069400.0 |
| 3.7095120000000001 | 852309.0 | 444312.0 | 1032130.0 |
| 3.7018680000000002 | 809498.0 | 437775.0 | 941328.0 |
| 3.694223 | 712415.0 | 411273.0 | 832435.0 |
| 3.6865790000000001 | 605453.0 | 401565.0 | 749147.0 |
| 3.6789350000000001 | 541873.0 | 393534.0 | 721033.0 |
| 3.6712899999999999 | 543540.0 | 386728.0 | 749267.0 |
| 3.663646 | 591161.0 | 396788.0 | 816455.0 |
| 3.6560009999999998 | 684528.0 | 397003.0 | 915964.0 |
| 3.6483569999999999 | 844384.0 | 411205.0 | 1050960.0 |
| 3.6407120000000002 | 1033920.0 | 409193.0 | 1190050.0 |
| 3.6330680000000002 | 1147800.0 | 413614.0 | 1256710.0 |
| 3.6254240000000002 | 1113240.0 | 396003.0 | 1193640.0 |
| 3.6177790000000001 | 955445.0 | 392494.0 | 1028650.0 |
| 3.6101350000000001 | 764839.0 | 369576.0 | 859311.0 |
| 3.60249 | 631616.0 | 369349.0 | 771339.0 |
| 3.594846 | 588009.0 | 357044.0 | 781147.0 |
| 3.587202 | 660919.0 | 365619.0 | 870949.0 |
| 3.5795569999999999 | 879075.0 | 361922.0 | 1048570.0 |
| 3.5719129999999999 | 1186520.0 | 368686.0 | 1286420.0 |
| 3.5642680000000002 | 1464850.0 | 360415.0 | 1485350.0 |
| 3.5566239999999998 | 1622390.0 | 349445.0 | 1559930.0 |
| 3.5489799999999998 | 1619610.0 | 331308.0 | 1491130.0 |
| 3.5413350000000001 | 1466990.0 | 301110.0 | 1324150.0 |
| 3.5336910000000001 | 1231620.0 | 282480.0 | 1125570.0 |
| 3.526046 | 988429.0 | 246199.0 | 942951.0 |
| 3.518402 | 790992.0 | 236954.0 | 800194.0 |
| 3.5107569999999999 | 668601.0 | 203809.0 | 711440.0 |
| 3.5031129999999999 | 610217.0 | 205479.0 | 671994.0 |
| 3.4954689999999999 | 582107.0 | 175074.0 | 653716.0 |
| 3.4878239999999998 | 567584.0 | 181190.0 | 643755.0 |
| 3.4801799999999998 | 560346.0 | 151698.0 | 641749.0 |
| 3.4725350000000001 | 555270.0 | 159765.0 | 641787.0 |
| 3.4648910000000002 | 545381.0 | 133764.0 | 638204.0 |
| 3.4572470000000002 | 525913.0 | 141732.0 | 623569.0 |
| 3.4496020000000001 | 499446.0 | 123074.0 | 596467.0 |
| 3.4419580000000001 | 469323.0 | 129832.0 | 561659.0 |
| 3.4343129999999999 | 436851.0 | 120985.0 | 526152.0 |
| 3.426669 | 402632.0 | 124688.0 | 494957.0 |
| 3.4190239999999998 | 368312.0 | 122808.0 | 470527.0 |
| 3.4113799999999999 | 337096.0 | 123515.0 | 454328.0 |
| 3.4037359999999999 | 311577.0 | 124860.0 | 445985.0 |
| 3.3960910000000002 | 294434.0 | 123699.0 | 440981.0 |
| 3.3884470000000002 | 285465.0 | 127231.0 | 439003.0 |
| 3.3808020000000001 | 280566.0 | 125776.0 | 441851.0 |
| 3.3731580000000001 | 275998.0 | 131391.0 | 448756.0 |
| 3.3655140000000001 | 271540.0 | 126612.0 | 461403.0 |
| 3.357869 | 270145.0 | 126063.0 | 476111.0 |
| 3.350225 | 264513.0 | 96220.6 | 479001.0 |
| 3.3425799999999999 | 242495.0 | 94381.2 | 457696.0 |
| 3.3349359999999999 | 201932.0 | 86738.8 | 416599.0 |
| 3.3272910000000002 | 155360.0 | 94406.5 | 374854.0 |
| 3.3196469999999998 | 121859.0 | 118037.0 | 350593.0 |
| 3.3120029999999998 | 112641.0 | 141505.0 | 348781.0 |
| 3.3043580000000001 | 123938.0 | 203664.0 | 365759.0 |
| 3.2967140000000001 | 145141.0 | 243946.0 | 395671.0 |
| 3.289069 | 182476.0 | 339867.0 | 438533.0 |
| 3.281425 | 237161.0 | 390975.0 | 491520.0 |
| 3.2737810000000001 | 289397.0 | 511286.0 | 537713.0 |
| 3.2661359999999999 | 317815.0 | 566716.0 | 560073.0 |
| 3.2584919999999999 | 357462.0 | 716006.0 | 570244.0 |
| 3.2508469999999998 | 463626.0 | 779136.0 | 638739.0 |
| 3.2432029999999998 | 568529.0 | 970140.0 | 807140.0 |
| 3.2355589999999999 | 790194.0 | 1021360.0 | 1089150.0 |
| 3.2279140000000002 | 1287400.0 | 1167450.0 | 1565240.0 |
| 3.2202700000000002 | 1911430.0 | 1157920.0 | 2125990.0 |
| 3.2126250000000001 | 2297070.0 | 1152410.0 | 2469120.0 |
| 3.2049810000000001 | 2275030.0 | 1065630.0 | 2382180.0 |
| 3.197336 | 1938730.0 | 934912.0 | 1955450.0 |
| 3.189692 | 1478670.0 | 813650.0 | 1475350.0 |
| 3.182048 | 1072650.0 | 667800.0 | 1136030.0 |
| 3.1744029999999999 | 780145.0 | 550461.0 | 919475.0 |
| 3.1667589999999999 | 576346.0 | 442662.0 | 728251.0 |
| 3.1591140000000002 | 450022.0 | 364232.0 | 570254.0 |
| 3.1514700000000002 | 367776.0 | 297811.0 | 492769.0 |
| 3.1438259999999998 | 308995.0 | 254280.0 | 451659.0 |
| 3.1361810000000001 | 268780.0 | 205873.0 | 427636.0 |
| 3.1285370000000001 | 244246.0 | 191789.0 | 416534.0 |
| 3.120892 | 230483.0 | 165673.0 | 412934.0 |
| 3.113248 | 221690.0 | 187222.0 | 411888.0 |
| 3.1056029999999999 | 216189.0 | 195951.0 | 413841.0 |
| 3.0979589999999999 | 213466.0 | 251677.0 | 420333.0 |
| 3.0903149999999999 | 217937.0 | 289113.0 | 433412.0 |
| 3.0826699999999998 | 234506.0 | 367684.0 | 457147.0 |
| 3.0750259999999998 | 286902.0 | 404045.0 | 513020.0 |
| 3.0673810000000001 | 454376.0 | 499339.0 | 653883.0 |
| 3.0597370000000002 | 666530.0 | 525012.0 | 901610.0 |
| 3.0520930000000002 | 893243.0 | 599473.0 | 1261050.0 |
| 3.044448 | 1397340.0 | 596575.0 | 1803710.0 |
| 3.0368040000000001 | 2205500.0 | 609839.0 | 2377290.0 |
| 3.0291589999999999 | 2874860.0 | 584607.0 | 2641430.0 |
| 3.021515 | 2990900.0 | 552282.0 | 2412910.0 |
| 3.0138699999999998 | 2566670.0 | 523195.0 | 1859600.0 |
| 3.0062259999999998 | 1894480.0 | 484172.0 | 1343140.0 |
| 2.9985819999999999 | 1293460.0 | 459840.0 | 1060930.0 |
| 2.9909370000000002 | 909414.0 | 424703.0 | 918422.0 |
| 2.9832930000000002 | 670689.0 | 404148.0 | 752351.0 |
| 2.9756480000000001 | 502141.0 | 384212.0 | 569440.0 |
| 2.9680040000000001 | 384278.0 | 370016.0 | 461809.0 |
| 2.9603600000000001 | 309551.0 | 370743.0 | 429125.0 |
| 2.952715 | 299207.0 | 360855.0 | 465235.0 |
| 2.945071 | 339839.0 | 374370.0 | 537318.0 |
| 2.9374259999999999 | 396998.0 | 361957.0 | 602396.0 |
| 2.9297819999999999 | 443259.0 | 376406.0 | 633427.0 |
| 2.9221370000000002 | 457109.0 | 359787.0 | 617154.0 |
| 2.9144929999999998 | 432891.0 | 371043.0 | 566831.0 |
| 2.9068489999999998 | 378473.0 | 354962.0 | 505588.0 |
| 2.8992040000000001 | 313635.0 | 360340.0 | 450092.0 |
| 2.8915600000000001 | 259043.0 | 349870.0 | 406936.0 |
| 2.883915 | 218530.0 | 355885.0 | 372492.0 |
| 2.876271 | 184997.0 | 354403.0 | 343494.0 |
| 2.868627 | 158717.0 | 360222.0 | 325227.0 |
| 2.8609819999999999 | 149615.0 | 364677.0 | 323354.0 |
| 2.8533379999999999 | 158472.0 | 367238.0 | 333284.0 |
| 2.8456929999999998 | 173292.0 | 373261.0 | 342189.0 |
| 2.8380489999999998 | 182769.0 | 372836.0 | 341714.0 |
| 2.8304049999999998 | 185606.0 | 377767.0 | 334880.0 |
| 2.8227600000000002 | 191151.0 | 379044.0 | 331462.0 |
| 2.8151160000000002 | 199151.0 | 380562.0 | 335725.0 |
| 2.807471 | 208486.0 | 386471.0 | 344082.0 |
| 2.7998270000000001 | 231208.0 | 380979.0 | 355997.0 |
| 2.7921819999999999 | 265836.0 | 389052.0 | 364590.0 |
| 2.784538 | 290465.0 | 376775.0 | 362598.0 |
| 2.776894 | 274574.0 | 383608.0 | 346969.0 |
| 2.7692489999999998 | 229347.0 | 365004.0 | 328861.0 |
| 2.7616049999999999 | 206175.0 | 369297.0 | 327174.0 |
| 2.7539600000000002 | 224555.0 | 345955.0 | 339014.0 |
| 2.7463160000000002 | 246966.0 | 347974.0 | 341355.0 |
| 2.7386720000000002 | 232660.0 | 323572.0 | 318570.0 |
| 2.7310270000000001 | 182661.0 | 324958.0 | 280737.0 |
| 2.7233830000000001 | 121630.0 | 304281.0 | 251937.0 |
| 2.715738 | 66221.7 | 304256.0 | 238712.0 |
| 2.708094 | 28912.8 | 288312.0 | 237359.0 |
| 2.7004489999999999 | 16045.2 | 283233.0 | 242467.0 |
| 2.6928049999999999 | 29188.7 | 273216.0 | 253854.0 |
| 2.6851609999999999 | 43285.1 | 261492.0 | 268256.0 |
| 2.6775159999999998 | 56184.6 | 257076.0 | 285536.0 |
| 2.6698719999999998 | 89936.3 | 241818.0 | 308784.0 |
| 2.6622270000000001 | 152923.0 | 241635.0 | 335219.0 |
| 2.6545830000000001 | 216209.0 | 226544.0 | 354740.0 |
| 2.6469390000000002 | 236236.0 | 229538.0 | 358653.0 |
| 2.639294 | 220035.0 | 217726.0 | 354894.0 |
| 2.63165 | 214128.0 | 218916.0 | 360278.0 |
| 2.6240049999999999 | 243449.0 | 213779.0 | 371353.0 |
| 2.6163609999999999 | 281121.0 | 213008.0 | 368984.0 |
| 2.6087159999999998 | 292956.0 | 215060.0 | 345801.0 |
| 2.6010719999999998 | 279949.0 | 216362.0 | 318321.0 |
| 2.5934279999999998 | 258913.0 | 222307.0 | 306748.0 |
| 2.5857830000000002 | 243144.0 | 230202.0 | 319754.0 |
| 2.5781390000000002 | 230917.0 | 237568.0 | 351107.0 |
| 2.5704940000000001 | 216704.0 | 250343.0 | 387540.0 |
| 2.5628500000000001 | 205609.0 | 252501.0 | 421497.0 |
| 2.5552060000000001 | 201590.0 | 267709.0 | 446489.0 |
| 2.547561 | 204212.0 | 267662.0 | 459641.0 |
| 2.539917 | 216829.0 | 283765.0 | 458704.0 |
| 2.5322719999999999 | 224110.0 | 284394.0 | 437796.0 |
| 2.5246279999999999 | 221051.0 | 301691.0 | 400605.0 |
| 2.5169839999999999 | 230859.0 | 297773.0 | 361625.0 |
| 2.5093390000000002 | 261500.0 | 321443.0 | 337580.0 |
| 2.5016949999999998 | 287642.0 | 317172.0 | 337667.0 |
| 2.4940500000000001 | 291514.0 | 344488.0 | 360094.0 |
| 2.4864060000000001 | 282311.0 | 352860.0 | 408607.0 |
| 2.478761 | 276607.0 | 373519.0 | 481457.0 |
| 2.471117 | 309549.0 | 397559.0 | 569998.0 |
| 2.463473 | 383767.0 | 400606.0 | 641520.0 |
| 2.4558279999999999 | 457749.0 | 425880.0 | 670794.0 |
| 2.4481839999999999 | 480329.0 | 406000.0 | 651951.0 |
| 2.4405389999999998 | 459963.0 | 419237.0 | 606480.0 |
| 2.4328949999999998 | 419547.0 | 386104.0 | 549296.0 |
| 2.4252509999999998 | 374136.0 | 390211.0 | 487218.0 |
| 2.4176060000000001 | 331774.0 | 364755.0 | 425174.0 |
| 2.4099620000000002 | 302831.0 | 381290.0 | 388410.0 |
| 2.402317 | 285926.0 | 377071.0 | 396046.0 |
| 2.3946730000000001 | 290570.0 | 413693.0 | 444422.0 |
| 2.3870279999999999 | 350012.0 | 427303.0 | 524538.0 |
| 2.3793839999999999 | 463274.0 | 461843.0 | 622429.0 |
| 2.37174 | 600557.0 | 486267.0 | 729542.0 |
| 2.3640949999999998 | 746776.0 | 495017.0 | 837186.0 |
| 2.3564509999999999 | 873233.0 | 524328.0 | 909749.0 |
| 2.3488060000000002 | 934528.0 | 509595.0 | 915505.0 |
| 2.3411620000000002 | 899746.0 | 540508.0 | 843693.0 |
| 2.3335180000000002 | 787911.0 | 522191.0 | 726645.0 |
| 2.3258730000000001 | 635682.0 | 554887.0 | 604694.0 |
| 2.3182290000000001 | 488203.0 | 543988.0 | 516506.0 |
| 2.310584 | 395047.0 | 577656.0 | 488584.0 |
| 2.30294 | 397575.0 | 570587.0 | 525377.0 |
| 2.2952949999999999 | 491999.0 | 599027.0 | 610201.0 |
| 2.2876509999999999 | 614070.0 | 590165.0 | 691363.0 |
| 2.2800069999999999 | 692352.0 | 609084.0 | 726266.0 |
| 2.2723620000000002 | 702840.0 | 597017.0 | 716067.0 |
| 2.2647179999999998 | 664253.0 | 607111.0 | 689089.0 |
| 2.2570730000000001 | 607638.0 | 599486.0 | 664873.0 |
| 2.2494290000000001 | 558809.0 | 607455.0 | 638486.0 |
| 2.2417850000000001 | 531897.0 | 605337.0 | 609200.0 |
| 2.23414 | 529182.0 | 615339.0 | 588628.0 |
| 2.226496 | 542285.0 | 612466.0 | 583144.0 |
| 2.2188509999999999 | 557163.0 | 622936.0 | 583920.0 |
| 2.2112069999999999 | 566101.0 | 617292.0 | 586386.0 |
| 2.2035619999999998 | 576102.0 | 631274.0 | 600832.0 |
| 2.1959179999999998 | 590001.0 | 632103.0 | 631852.0 |
| 2.1882739999999998 | 610776.0 | 652513.0 | 677334.0 |
| 2.1806290000000002 | 644935.0 | 660921.0 | 722287.0 |
| 2.1729850000000002 | 679826.0 | 687614.0 | 742227.0 |
| 2.16534 | 697121.0 | 699690.0 | 731850.0 |
| 2.1576960000000001 | 691498.0 | 725613.0 | 708781.0 |
| 2.1500520000000001 | 683128.0 | 742744.0 | 702231.0 |
| 2.142407 | 694083.0 | 764426.0 | 728088.0 |
| 2.134763 | 736417.0 | 790975.0 | 781055.0 |
| 2.1271179999999998 | 805249.0 | 808192.0 | 839661.0 |
| 2.1194739999999999 | 872953.0 | 844951.0 | 881705.0 |
| 2.1118299999999999 | 912205.0 | 858698.0 | 902889.0 |
| 2.1041850000000002 | 914315.0 | 900594.0 | 907749.0 |
| 2.0965410000000002 | 903594.0 | 909575.0 | 911650.0 |
| 2.0888960000000001 | 917672.0 | 941562.0 | 935749.0 |
| 2.0812520000000001 | 949215.0 | 942144.0 | 969130.0 |
| 2.073607 | 984824.0 | 954938.0 | 1011310.0 |
| 2.065963 | 1053350.0 | 945126.0 | 1091490.0 |
| 2.058319 | 1196810.0 | 941395.0 | 1205010.0 |
| 2.0506739999999999 | 1483450.0 | 921529.0 | 1339990.0 |
| 2.0430299999999999 | 1693970.0 | 904650.0 | 1436080.0 |
| 2.0353850000000002 | 1955020.0 | 876446.0 | 1550180.0 |
| 2.0277409999999998 | 2579730.0 | 845746.0 | 1763820.0 |
| 2.0200969999999998 | 3408640.0 | 811763.0 | 1982550.0 |
| 2.0124520000000001 | 3849460.0 | 769930.0 | 2008460.0 |
| 2.0048080000000001 | 3606660.0 | 733305.0 | 1764160.0 |
| 1.997163 | 2881920.0 | 686036.0 | 1397570.0 |
| 1.989519 | 2037610.0 | 648943.0 | 1102270.0 |
| 1.9818750000000001 | 1384100.0 | 603600.0 | 929052.0 |
| 1.9742310000000001 | 992406.0 | 567258.0 | 814071.0 |
| 1.9665859999999999 | 753848.0 | 527291.0 | 692276.0 |
| 1.958942 | 623612.0 | 494013.0 | 604405.0 |
| 1.951298 | 540418.0 | 459314.0 | 552877.0 |
| 1.9436530000000001 | 473702.0 | 430690.0 | 515875.0 |
| 1.9360090000000001 | 427092.0 | 400396.0 | 489629.0 |
| 1.9283650000000001 | 399402.0 | 377013.0 | 470651.0 |
| 1.92072 | 381618.0 | 352995.0 | 454713.0 |
| 1.913076 | 368600.0 | 333082.0 | 437514.0 |
| 1.905432 | 357336.0 | 315860.0 | 417908.0 |
| 1.897788 | 346192.0 | 298041.0 | 398551.0 |
| 1.8901429999999999 | 330191.0 | 287406.0 | 379786.0 |
| 1.8824989999999999 | 312262.0 | 270673.0 | 362925.0 |
| 1.8748549999999999 | 292595.0 | 265076.0 | 347953.0 |
| 1.86721 | 272617.0 | 249782.0 | 334639.0 |
| 1.8595660000000001 | 254000.0 | 247843.0 | 322334.0 |
| 1.8519220000000001 | 239224.0 | 233887.0 | 310155.0 |
| 1.8442780000000001 | 231139.0 | 235331.0 | 299353.0 |
| 1.836633 | 231598.0 | 222673.0 | 293294.0 |
| 1.828989 | 238261.0 | 228407.0 | 291366.0 |
| 1.821345 | 246828.0 | 217112.0 | 292239.0 |
| 1.8137000000000001 | 253521.0 | 225929.0 | 293718.0 |
| 1.8060560000000001 | 258608.0 | 216336.0 | 295268.0 |
| 1.7984119999999999 | 261355.0 | 226361.0 | 296459.0 |
| 1.790767 | 261232.0 | 219271.0 | 298819.0 |
| 1.783123 | 260445.0 | 227826.0 | 304370.0 |
| 1.775479 | 265388.0 | 222915.0 | 313368.0 |
| 1.767835 | 277400.0 | 229081.0 | 322124.0 |
| 1.7601899999999999 | 287905.0 | 226151.0 | 325453.0 |
| 1.7525459999999999 | 290215.0 | 230196.0 | 321955.0 |
| 1.744902 | 283876.0 | 230457.0 | 316164.0 |
| 1.7372570000000001 | 276258.0 | 232967.0 | 314925.0 |
| 1.7296130000000001 | 273867.0 | 238373.0 | 320500.0 |
| 1.7219690000000001 | 277949.0 | 238644.0 | 329790.0 |
| 1.714324 | 283929.0 | 248173.0 | 338476.0 |
| 1.70668 | 290463.0 | 246051.0 | 345098.0 |
| 1.699036 | 297526.0 | 256984.0 | 351108.0 |
| 1.691392 | 299992.0 | 254048.0 | 354668.0 |
| 1.6837470000000001 | 296186.0 | 262796.0 | 355297.0 |
| 1.6761029999999999 | 286924.0 | 261231.0 | 353979.0 |
| 1.6684589999999999 | 274730.0 | 266989.0 | 351799.0 |
| 1.660814 | 265709.0 | 266116.0 | 351029.0 |
| 1.65317 | 260215.0 | 270640.0 | 348207.0 |
| 1.645526 | 251070.0 | 267419.0 | 339697.0 |
| 1.6378820000000001 | 236172.0 | 272360.0 | 326117.0 |
| 1.6302369999999999 | 221645.0 | 269667.0 | 313402.0 |
| 1.622593 | 211711.0 | 277909.0 | 305014.0 |
| 1.614949 | 208690.0 | 278187.0 | 300578.0 |
| 1.6073040000000001 | 210151.0 | 290285.0 | 298412.0 |
| 1.5996600000000001 | 213441.0 | 294516.0 | 295600.0 |
| 1.5920160000000001 | 216653.0 | 310262.0 | 292400.0 |
| 1.584371 | 218359.0 | 314959.0 | 288709.0 |
| 1.576727 | 217610.0 | 332465.0 | 282909.0 |
| 1.569083 | 214439.0 | 340320.0 | 274635.0 |
| 1.561439 | 209604.0 | 363639.0 | 265361.0 |
| 1.5537939999999999 | 207903.0 | 376499.0 | 259490.0 |
| 1.5461499999999999 | 212089.0 | 406878.0 | 257492.0 |
| 1.5385059999999999 | 219754.0 | 428279.0 | 258259.0 |
| 1.530861 | 227608.0 | 462733.0 | 260167.0 |
| 1.523217 | 238528.0 | 484415.0 | 264747.0 |
| 1.5155730000000001 | 251993.0 | 514698.0 | 272380.0 |
| 1.5079279999999999 | 263046.0 | 523072.0 | 278356.0 |
| 1.500284 | 266517.0 | 534446.0 | 277385.0 |
| 1.49264 | 262983.0 | 520039.0 | 270994.0 |
| 1.484996 | 258786.0 | 565173.0 | 266891.0 |
| 1.4773510000000001 | 257663.0 | 590754.0 | 269508.0 |
| 1.4697070000000001 | 261490.0 | 677052.0 | 277364.0 |
| 1.4620629999999999 | 270697.0 | 754805.0 | 288011.0 |
| 1.454418 | 281634.0 | 865665.0 | 298667.0 |
| 1.446774 | 289508.0 | 986664.0 | 306482.0 |
| 1.43913 | 290904.0 | 1045340.0 | 309700.0 |
| 1.431486 | 284343.0 | 1200920.0 | 309152.0 |
| 1.4238409999999999 | 275175.0 | 1265340.0 | 306703.0 |
| 1.4161969999999999 | 269914.0 | 1489460.0 | 306028.0 |
| 1.4085529999999999 | 266523.0 | 1582650.0 | 307346.0 |
| 1.400908 | 264082.0 | 1904150.0 | 313575.0 |
| 1.3932640000000001 | 264954.0 | 2042380.0 | 334679.0 |
| 1.3856200000000001 | 267305.0 | 2474570.0 | 364868.0 |
| 1.3779749999999999 | 267491.0 | 2570770.0 | 411616.0 |
| 1.370331 | 269508.0 | 3007190.0 | 476863.0 |
| 1.362687 | 276618.0 | 2990430.0 | 533825.0 |
| 1.355043 | 278462.0 | 3302300.0 | 540657.0 |
| 1.3473980000000001 | 264313.0 | 3284730.0 | 484886.0 |
| 1.3397539999999999 | 233408.0 | 3487000.0 | 402460.0 |
| 1.3321099999999999 | 203432.0 | 3659950.0 | 350329.0 |
| 1.324465 | 179647.0 | 3846190.0 | 340731.0 |
| 1.316821 | 165490.0 | 4093550.0 | 387567.0 |
| 1.309177 | 168655.0 | 4231000.0 | 481707.0 |
| 1.3015330000000001 | 184998.0 | 4276940.0 | 581089.0 |
| 1.2938879999999999 | 200112.0 | 4277190.0 | 622869.0 |
| 1.2862439999999999 | 200260.0 | 4064920.0 | 586634.0 |
| 1.2786 | 183684.0 | 3914060.0 | 508884.0 |
| 1.2709550000000001 | 159188.0 | 3622410.0 | 440266.0 |
| 1.2633110000000001 | 138233.0 | 3403200.0 | 395680.0 |
| 1.2556670000000001 | 126878.0 | 3142820.0 | 358202.0 |
| 1.248022 | 123903.0 | 2927980.0 | 317788.0 |
| 1.240378 | 125660.0 | 2710580.0 | 290111.0 |
| 1.232734 | 125619.0 | 2523630.0 | 267683.0 |
| 1.22509 | 123433.0 | 2302940.0 | 247719.0 |
| 1.2174450000000001 | 123625.0 | 2126440.0 | 228722.0 |
| 1.2098009999999999 | 124000.0 | 1925750.0 | 209998.0 |
| 1.2021569999999999 | 122209.0 | 1769650.0 | 193431.0 |
| 1.194512 | 115155.0 | 1628690.0 | 178355.0 |
| 1.186868 | 105651.0 | 1502610.0 | 164707.0 |
| 1.179224 | 97764.6 | 1431670.0 | 155172.0 |
| 1.1715789999999999 | 93388.0 | 1337430.0 | 150702.0 |
| 1.1639349999999999 | 88564.3 | 1283780.0 | 147657.0 |
| 1.156291 | 84479.9 | 1205740.0 | 144027.0 |
| 1.148647 | 85573.8 | 1158480.0 | 139828.0 |
| 1.1410020000000001 | 87761.5 | 1091180.0 | 136176.0 |
| 1.1333580000000001 | 90131.3 | 1045140.0 | 133043.0 |
| 1.1257140000000001 | 92725.8 | 986729.0 | 129972.0 |
| 1.118069 | 98864.0 | 950339.0 | 129073.0 |
| 1.110425 | 106239.0 | 904045.0 | 131970.0 |
| 1.102781 | 113640.0 | 877897.0 | 136041.0 |
| 1.095137 | 118476.0 | 844151.0 | 137541.0 |
| 1.0874919999999999 | 118842.0 | 828728.0 | 136369.0 |
| 1.0798479999999999 | 118702.0 | 807224.0 | 135355.0 |
| 1.0722039999999999 | 119965.0 | 803366.0 | 136276.0 |
| 1.064559 | 124160.0 | 793801.0 | 138465.0 |
| 1.056915 | 134351.0 | 801815.0 | 142014.0 |
| 1.0492710000000001 | 149793.0 | 803238.0 | 147319.0 |
| 1.0416259999999999 | 166598.0 | 823626.0 | 153418.0 |
| 1.033982 | 182736.0 | 835815.0 | 160053.0 |
| 1.026338 | 199963.0 | 869241.0 | 167907.0 |
| 1.018694 | 221511.0 | 892813.0 | 179339.0 |
| 1.0110490000000001 | 244921.0 | 939446.0 | 192539.0 |
| 1.0034050000000001 | 269626.0 | 974127.0 | 206536.0 |
| 0.99576100000000001 | 295043.0 | 1031390.0 | 221438.0 |
| 0.98811599999999999 | 321764.0 | 1074240.0 | 238001.0 |
| 0.98047200000000001 | 347005.0 | 1136740.0 | 256958.0 |
| 0.97282800000000003 | 373911.0 | 1185400.0 | 277827.0 |
| 0.96518300000000001 | 402439.0 | 1246070.0 | 299442.0 |
| 0.95753900000000003 | 426868.0 | 1296990.0 | 320231.0 |
| 0.94989500000000004 | 444864.0 | 1350370.0 | 338897.0 |
| 0.94225099999999995 | 455490.0 | 1397620.0 | 355749.0 |
| 0.93460600000000005 | 460616.0 | 1438020.0 | 371982.0 |
| 0.92696199999999995 | 463754.0 | 1473430.0 | 389470.0 |
| 0.91931799999999997 | 465852.0 | 1496710.0 | 407530.0 |
| 0.91167299999999996 | 465498.0 | 1512950.0 | 424757.0 |
| 0.90402899999999997 | 463617.0 | 1515290.0 | 439938.0 |
| 0.89638499999999999 | 459334.0 | 1509480.0 | 452932.0 |
| 0.888741 | 447732.0 | 1489750.0 | 461842.0 |
| 0.88109599999999999 | 429176.0 | 1462710.0 | 465311.0 |
| 0.87345200000000001 | 406500.0 | 1422170.0 | 463867.0 |
| 0.86580800000000002 | 381214.0 | 1376810.0 | 459082.0 |
| 0.85816300000000001 | 354805.0 | 1320100.0 | 452240.0 |
| 0.85051900000000002 | 328813.0 | 1260360.0 | 444676.0 |
| 0.84287500000000004 | 305320.0 | 1195700.0 | 436330.0 |
| 0.83523000000000003 | 284031.0 | 1126660.0 | 426530.0 |
| 0.82758600000000004 | 262887.0 | 1062290.0 | 414661.0 |
| 0.81994199999999995 | 240260.0 | 991044.0 | 400118.0 |
| 0.81229799999999996 | 215584.0 | 930782.0 | 382121.0 |
| 0.80465299999999995 | 188163.0 | 863323.0 | 361571.0 |
| 0.79700899999999997 | 160386.0 | 807603.0 | 339683.0 |
| 0.78936499999999998 | 137999.0 | 747523.0 | 319001.0 |
| 0.78171999999999997 | 120904.0 | 697822.0 | 300851.0 |
| 0.77407599999999999 | 107707.0 | 645564.0 | 284112.0 |
| 0.766432 | 95080.7 | 603641.0 | 267150.0 |
| 0.75878800000000002 | 82549.7 | 559021.0 | 249777.0 |
| 0.75114300000000001 | 71321.5 | 525753.0 | 233446.0 |
| 0.74349900000000002 | 67322.2 | 488886.0 | 220965.0 |
| 0.73585500000000004 | 67154.0 | 462152.0 | 211511.0 |
| 0.72821000000000002 | 68117.2 | 432868.0 | 202638.0 |
| 0.72056600000000004 | 67882.8 | 408814.0 | 194125.0 |
| 0.71292199999999994 | 68345.8 | 385034.0 | 186077.0 |
| 0.70527700000000004 | 67894.9 | 362905.0 | 176566.0 |
| 0.69763299999999995 | 61197.7 | 342141.0 | 162410.0 |
| 0.68998899999999996 | 47688.9 | 323992.0 | 143289.0 |
| 0.68234499999999998 | 31666.3 | 307327.0 | 121817.0 |
| 0.67469999999999997 | 21190.4 | 294488.0 | 104939.0 |
| 0.66705599999999998 | 18087.8 | 283958.0 | 95290.0 |
| 0.659412 | 19569.1 | 274803.0 | 91061.3 |
| 0.65176699999999999 | 21526.6 | 269518.0 | 89655.6 |
| 0.644123 | 25340.6 | 262028.0 | 90776.6 |
| 0.63647900000000002 | 34214.6 | 260351.0 | 94184.6 |
| 0.628834 | 43906.4 | 251854.0 | 98109.5 |
| 0.62119000000000002 | 48660.4 | 251891.0 | 98734.2 |
| 0.61354600000000004 | 48103.0 | 242205.0 | 93732.4 |
| 0.60590200000000005 | 43225.8 | 242110.0 | 84568.0 |
| 0.59825700000000004 | 35721.4 | 233251.0 | 74803.2 |Lac
NAA
Cho
Gln+Glu
Glc
b
4
3
2
1
3
4
1
1
5
3
2
2
4
### Chart:
| Category | Glycine |
|---|---|
| Central 1 | 0.2924900381275794 |
| Central 2 | 0.2340972001912882 |
| Edge 3 | 0.0749957101549597 |
| Edge 4 | 0.057196201825315617 |
### Chart:
| Category | Glycine |
|---|---|
| Central 1 | 0.7987057007911983 |
| Central 2 | 0.8714143500776454 |
| Edge 3 | 0.3509919502880359 |
| Edge 4 | 0.3292570324710499 |
### Chart:
| Category | Serine |
|---|---|
| Central 1 | 0.00733480171691732 |
| Central 2 | 0.004483864534584588 |
| Edge 3 | 0.002581540258697722 |
| Edge 4 | 0.0016588549246926015 |
### Chart:
| Category | Serine |
|---|---|
| Central 1 | 0.17448441559329497 |
| Central 2 | 0.20922096845653096 |
| Central 3 | 0.09095808547391224 |
| Edge 4 | 0.007261213101318549 |
| Edge 5 | 0.013299687290823394 |
### Chart:
| Category | Serine |
|---|---|
| Central 1 | 0.008602513743140876 |
| Central 2 | 0.009744670041373396 |
| Edge 3 | 0.007504241766271697 |
| Edge 4 | 0.007522104549438816 |
### Chart:
| Category | Glycine |
|---|---|
| Central 1 | 8.421552638274465 |
| Central 2 | 7.290519098299148 |
| Central 3 | 2.8828955619430463 |
| Edge 4 | 0.25633871792332175 |
| Edge 5 | 0.43689890171732887 |

## Slide 2
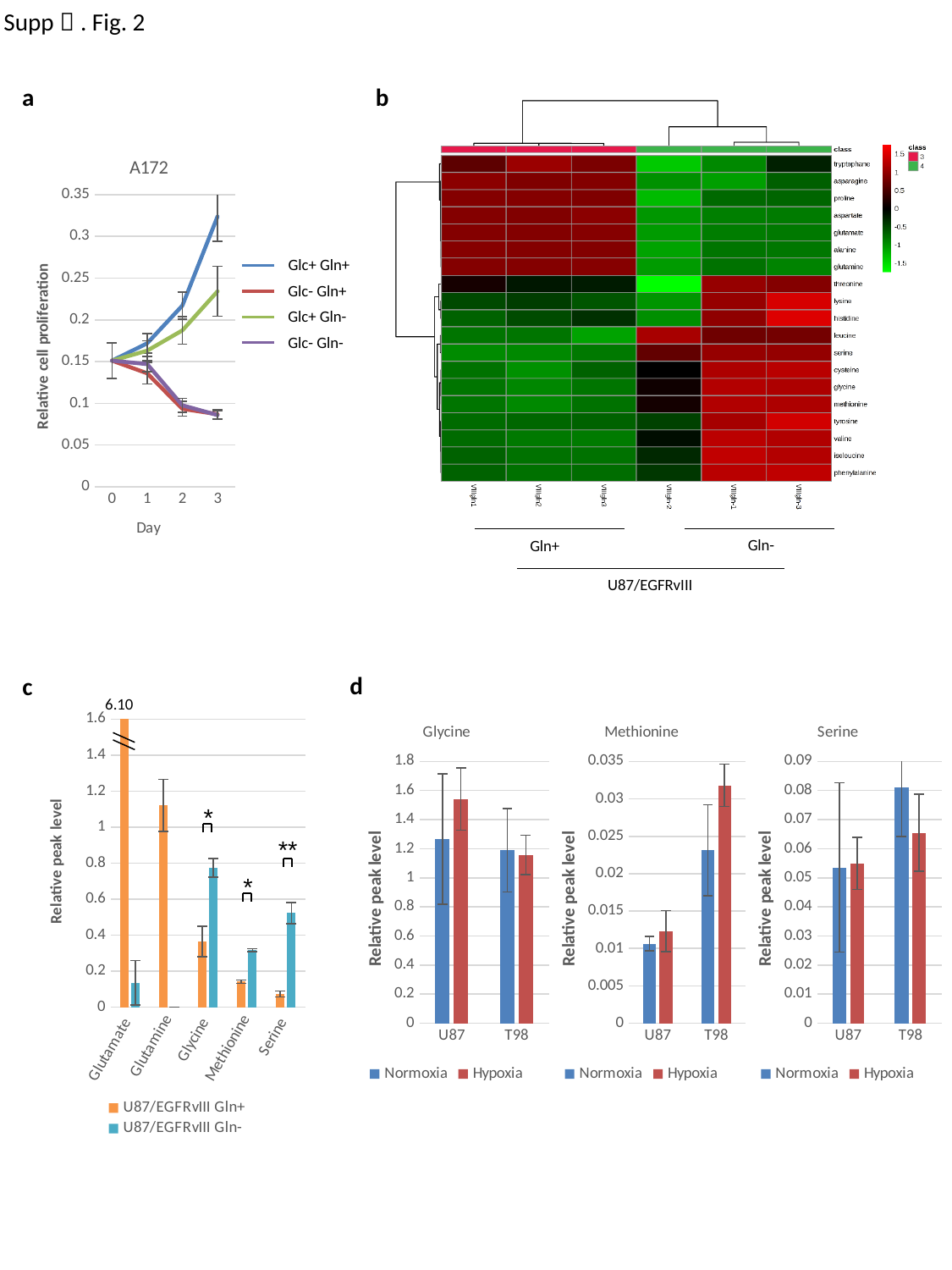

Suppｌ. Fig. 2
a
b
### Chart: A172
| Category | Glc+/Gln+ | Glc-/Gln+ | Glc+/Gln- | Glc-/Gln- |
|---|---|---|---|---|
| 0 | 0.15103333333333332 | 0.15103333333333332 | 0.15103333333333332 | 0.15103333333333332 |
| 1 | 0.17179999999999998 | 0.13588333333333333 | 0.16301666666666667 | 0.1469 |
| 2 | 0.21708333333333332 | 0.09326666666666668 | 0.1873166666666667 | 0.09755000000000001 |
| 3 | 0.32383333333333336 | 0.08683333333333333 | 0.23418333333333333 | 0.08576666666666666 |Glc+ Gln+
Glc- Gln+
Glc+ Gln-
Glc- Gln-
Gln-
Gln+
U87/EGFRvIII
d
### Chart
| Category | U87/EGFRvIII | U87/EGFRvIII |
|---|---|---|
| Glutamate | 6.104431939172218 | 0.136160374658276 |
| Glutamine | 1.12117810071803 | 0.0016975760233336973 |
| Glycine | 0.36534837653161517 | 0.7739154130082633 |
| Methionine | 0.14214598842227255 | 0.31747682728476395 |
| Serine | 0.07390855746969531 | 0.5230846908148593 |c
6.10
### Chart: Glycine
| Category | Normoxia | Hypoxia |
|---|---|---|
| U87 | 1.2667481208931246 | 1.5413926604649018 |
| T98 | 1.1896434599138994 | 1.1573213807108909 |
### Chart: Methionine
| Category | Normoxia | Hypoxia |
|---|---|---|
| U87 | 0.010642070978868453 | 0.01233266499490359 |
| T98 | 0.023141894740014702 | 0.031803510282343815 |
### Chart: Serine
| Category | Normoxia | Hypoxia |
|---|---|---|
| U87 | 0.0535686402065808 | 0.05496107329789246 |
| T98 | 0.08106299748566267 | 0.06547915171347113 |*
**
*

## Slide 3
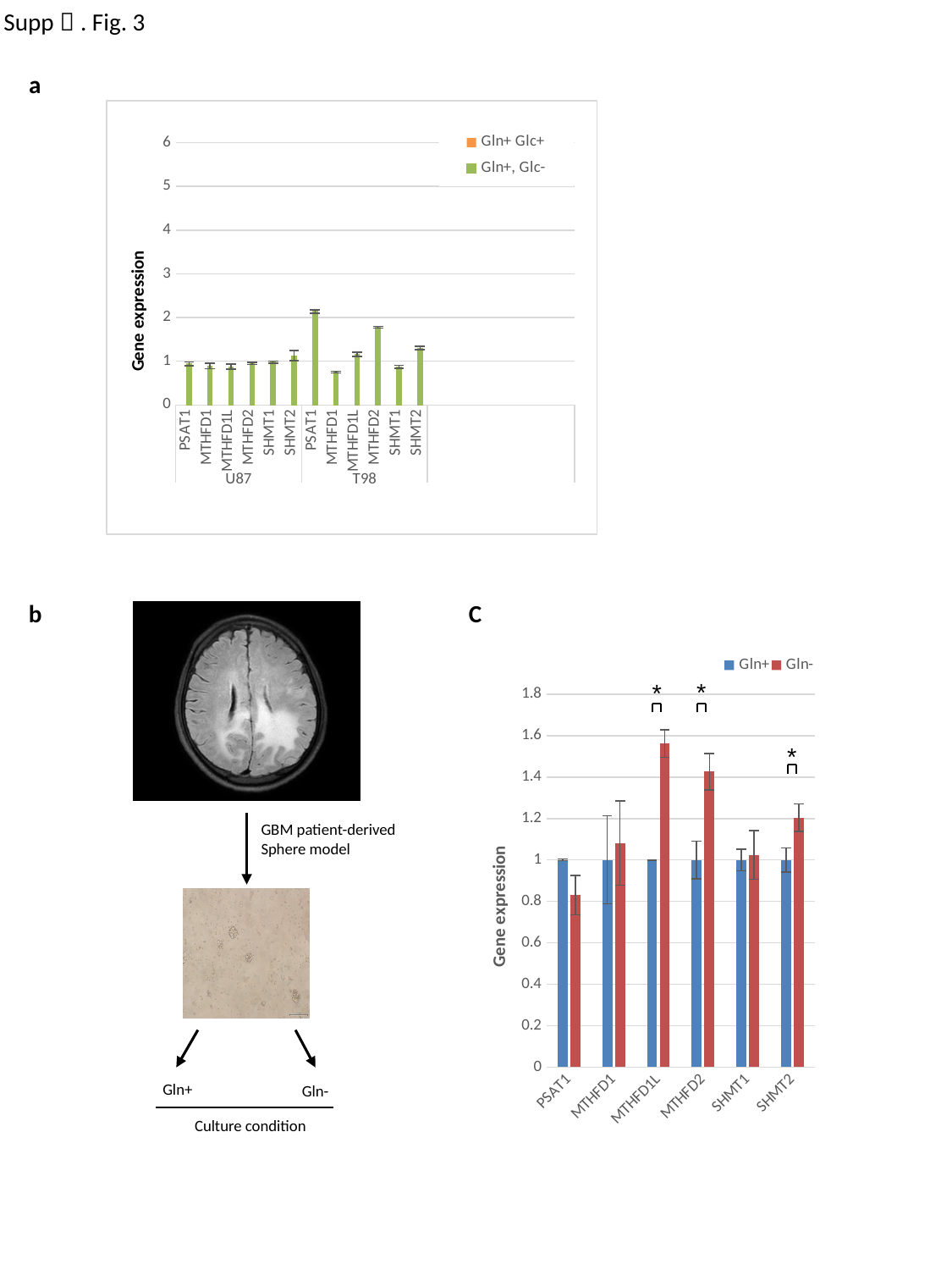

Suppｌ. Fig. 3
a
### Chart
| Category | Gln+ Glc+ | Gln+, Glc- |
|---|---|---|
| PSAT1 | 1.0 | 0.9440138782033308 |
| MTHFD1 | 1.0 | 0.893078999389241 |
| MTHFD1L | 1.0 | 0.876133257336469 |
| MTHFD2 | 1.0 | 0.9523676661532747 |
| SHMT1 | 1.0 | 0.9792763281850504 |
| SHMT2 | 1.0 | 1.1263967649616238 |
| PSAT1 | 1.0 | 2.1404097994779923 |
| MTHFD1 | 1.0 | 0.7519385205359572 |
| MTHFD1L | 1.0 | 1.1559643522706966 |
| MTHFD2 | 1.0 | 1.775831903440282 |
| SHMT1 | 1.0 | 0.869454342449261 |
| SHMT2 | 1.0 | 1.303458996056379 |b
C
### Chart
| Category | Gln+ | Gln- |
|---|---|---|
| PSAT1 | 1.0 | 0.8295937987971389 |
| MTHFD1 | 1.0 | 1.0817714224584816 |
| MTHFD1L | 1.0 | 1.5618857546179985 |
| MTHFD2 | 1.0 | 1.426677323944796 |
| SHMT1 | 1.0 | 1.0245738723086573 |
| SHMT2 | 1.0 | 1.2046344123843835 |*
*
*
GBM patient-derived
Sphere model
Gln+
Gln-
Culture condition

## Slide 4
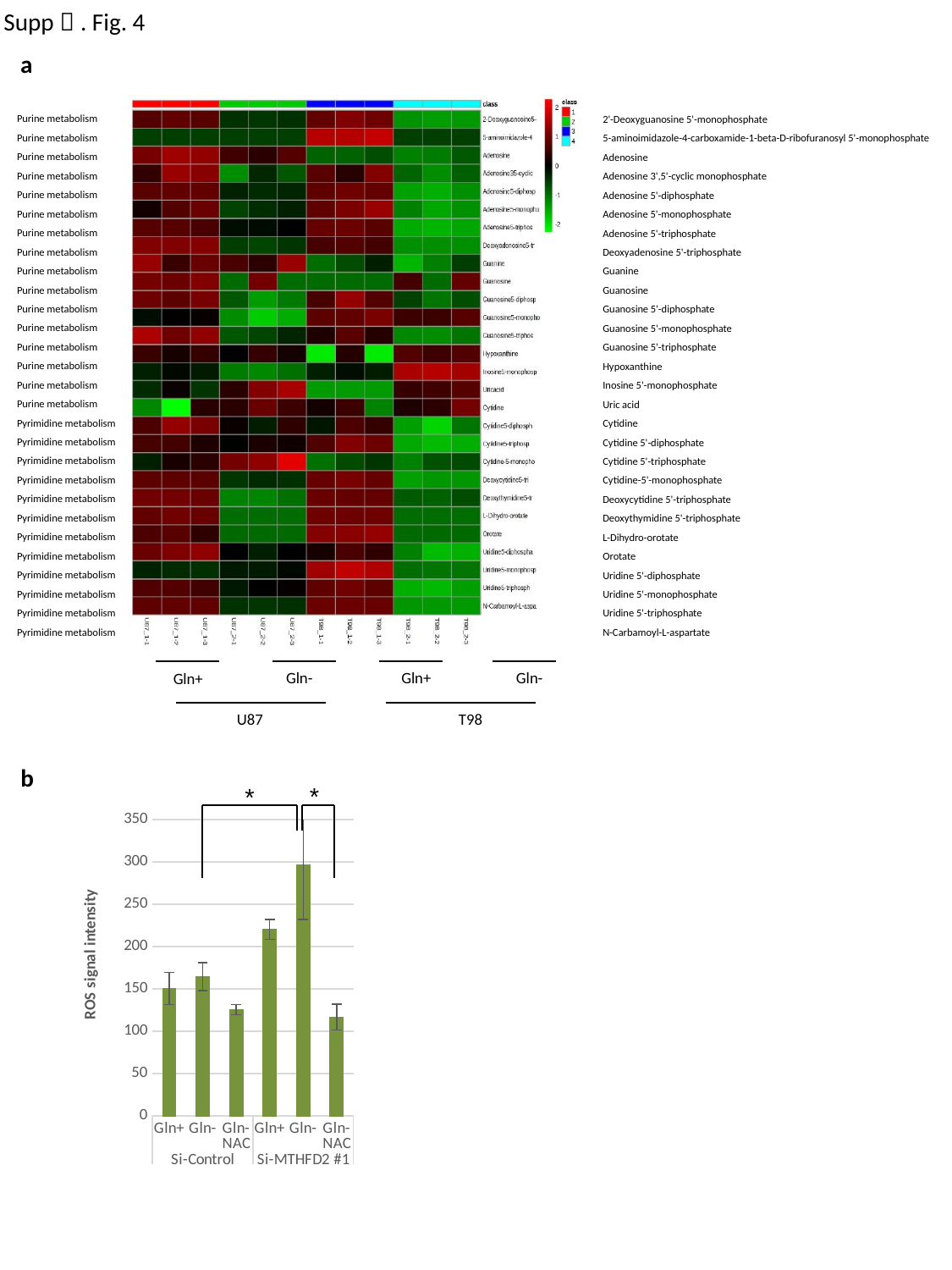

Suppｌ. Fig. 4
a
| Purine metabolism |
| --- |
| Purine metabolism |
| Purine metabolism |
| Purine metabolism |
| Purine metabolism |
| Purine metabolism |
| Purine metabolism |
| Purine metabolism |
| Purine metabolism |
| Purine metabolism |
| Purine metabolism |
| Purine metabolism |
| Purine metabolism |
| Purine metabolism |
| Purine metabolism |
| Purine metabolism |
| Pyrimidine metabolism |
| Pyrimidine metabolism |
| Pyrimidine metabolism |
| Pyrimidine metabolism |
| Pyrimidine metabolism |
| Pyrimidine metabolism |
| Pyrimidine metabolism |
| Pyrimidine metabolism |
| Pyrimidine metabolism |
| Pyrimidine metabolism |
| Pyrimidine metabolism |
| Pyrimidine metabolism |
| 2'-Deoxyguanosine 5'-monophosphate |
| --- |
| 5-aminoimidazole-4-carboxamide-1-beta-D-ribofuranosyl 5'-monophosphate |
| Adenosine |
| Adenosine 3',5'-cyclic monophosphate |
| Adenosine 5'-diphosphate |
| Adenosine 5'-monophosphate |
| Adenosine 5'-triphosphate |
| Deoxyadenosine 5'-triphosphate |
| Guanine |
| Guanosine |
| Guanosine 5'-diphosphate |
| Guanosine 5'-monophosphate |
| Guanosine 5'-triphosphate |
| Hypoxanthine |
| Inosine 5'-monophosphate |
| Uric acid |
| Cytidine |
| Cytidine 5'-diphosphate |
| Cytidine 5'-triphosphate |
| Cytidine-5'-monophosphate |
| Deoxycytidine 5'-triphosphate |
| Deoxythymidine 5'-triphosphate |
| L-Dihydro-orotate |
| Orotate |
| Uridine 5'-diphosphate |
| Uridine 5'-monophosphate |
| Uridine 5'-triphosphate |
| N-Carbamoyl-L-aspartate |
Gln-
Gln+
Gln-
Gln+
U87
T98
b
*
*
### Chart
| Category | |
|---|---|
| Gln+ | 150.66666666666666 |
| Gln- | 164.66666666666666 |
| Gln- NAC | 125.66666666666667 |
| Gln+ | 220.33333333333334 |
| Gln- | 296.6666666666667 |
| Gln- NAC | 116.66666666666667 |

## Slide 5
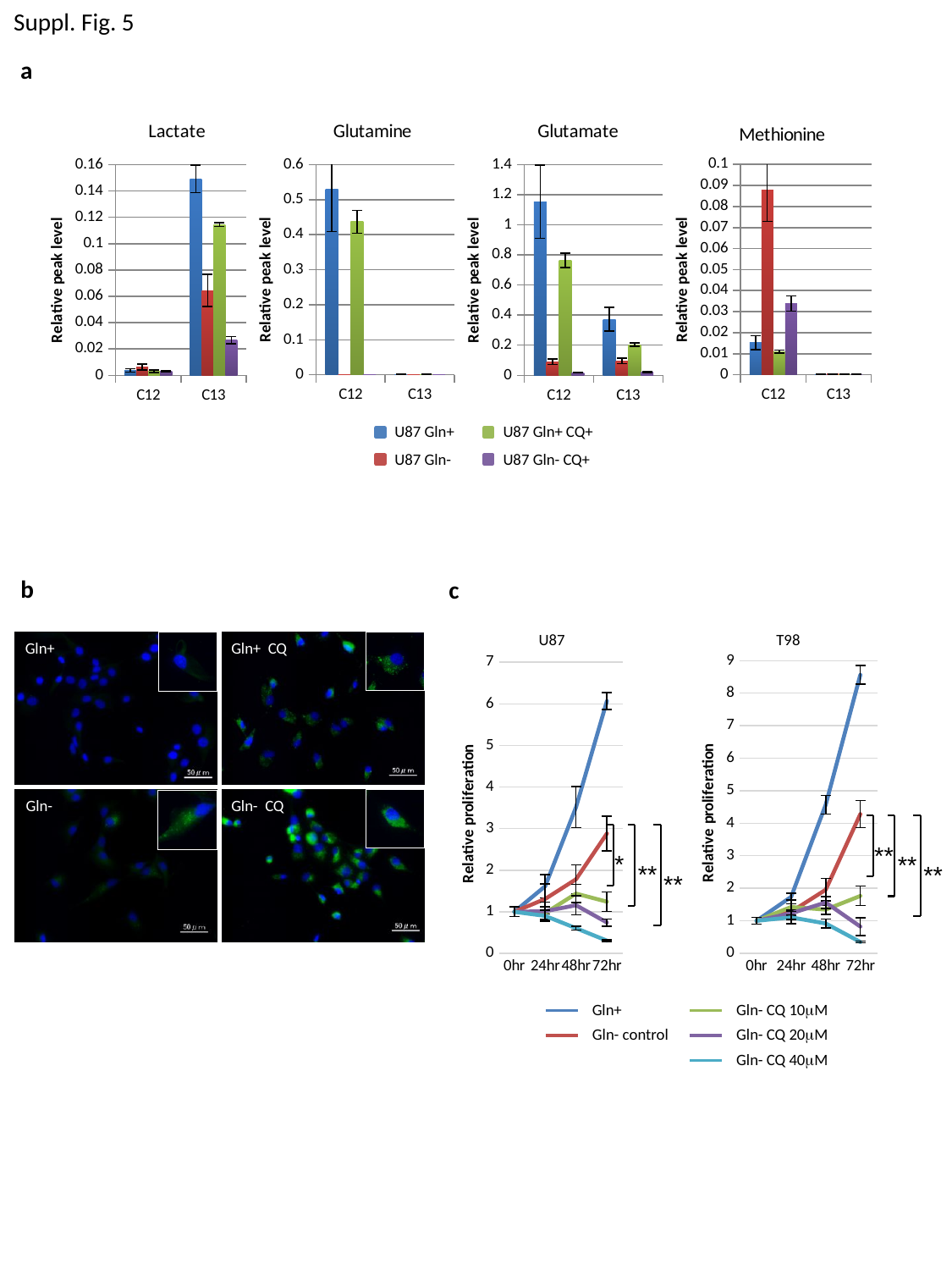

Suppl. Fig. 5
a
### Chart: Methionine
| Category | U87 G+ | U87 G- | U87 G+ CHL+ | U87 G- CHL+ |
|---|---|---|---|---|
| C12 | 0.015249527884204639 | 0.08777082130217691 | 0.011054489149670542 | 0.033856767310399195 |
| C13 | 0.00018083362825604642 | 0.00030912074970207366 | 0.00015803637932559447 | 0.00022747484045155142 |
### Chart: Lactate
| Category | U87 G+ | U87 G- | U87 G+ CHL+ | U87 G- CHL+ |
|---|---|---|---|---|
| C12 | 0.0037881260834109554 | 0.006296764022846642 | 0.0030153276141977907 | 0.003327105831373276 |
| C13 | 0.1491161330889465 | 0.06442005962485782 | 0.11450885227317564 | 0.026674045929737617 |
### Chart: Glutamine
| Category | U87 G+ | U87 G- | U87 G+ CHL+ | U87 G- CHL+ |
|---|---|---|---|---|
| C12 | 0.529091785528663 | 0.0009423934423756034 | 0.4368452566551267 | 0.0008119858976512855 |
| C13 | 0.0013196713252627433 | 1.2012140710101565e-05 | 0.0012893335332966255 | 0.00010391348838190854 |
### Chart: Glutamate
| Category | U87 G+ | U87 G- | U87 G+ CHL+ | U87 G- CHL+ |
|---|---|---|---|---|
| C12 | 1.152530988006765 | 0.0903876199498402 | 0.7646196535545053 | 0.016861630206210797 |
| C13 | 0.37299694811494405 | 0.096268931080987 | 0.20385571142061362 | 0.021281069605211107 |U87 Gln+
U87 Gln+ CQ+
U87 Gln-
U87 Gln- CQ+
b
c
U87
T98
Gln+
Gln+ CQ
Gln-
Gln- CQ
### Chart
| Category | Gln+ | Gln- control | Gln- Chl 10μM | Gln- Chl 20μM | Gln- Chl 40μM |
|---|---|---|---|---|---|
| 0hr | 1.0 | 1.0 | 1.0 | 1.0 | 1.0 |
| 24hr | 1.7343989443214711 | 1.2764558147454452 | 1.4249106078665075 | 1.230972245870935 | 1.106674612634088 |
| 48hr | 4.56942789034565 | 1.9541120381406436 | 1.3437340371190192 | 1.5625744934445769 | 0.9166099097565127 |
| 72hr | 8.563851523923038 | 4.280708326238719 | 1.7636216584369144 | 0.8217691128894942 | 0.3482887791588626 |
### Chart
| Category | Gln+ | Gln- control | Gln- Chl 10μM | Gln- Chl 20μM | Gln- Chl 40μM |
|---|---|---|---|---|---|
| 0hr | 1.0 | 1.0 | 1.0 | 1.0 | 1.0 |
| 24hr | 1.614379837817914 | 1.3088370807224476 | 0.9667803169922596 | 1.0146516771102103 | 0.9013545890158497 |
| 48hr | 3.518429782528566 | 1.7819756726870624 | 1.4378916328787321 | 1.1576667895318837 | 0.6049115370438629 |
| 72hr | 6.070217471433837 | 2.8826022852930335 | 1.2429045337265021 | 0.7418908956874309 | 0.30492075193512724 |**
*
**
**
**
**
Gln+
Gln- CQ 10mM
Gln- control
Gln- CQ 20mM
Gln- CQ 40mM
